# Supplementary figures and images for: Steady-state neuron-predominant LINE-1 encoded ORF1p protein and LINE-1 RNA increase with aging in the mouse and human brain
Source: eLife. 2025 Sep 25;13:RP100687. doi: 10.7554/eLife.100687 (PMC12463392; doi:10.7554/eLife.100687)

Figure 1 – Source data 1

H

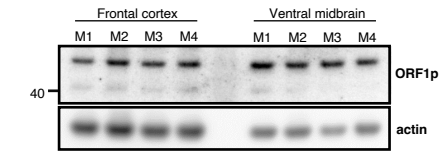

J

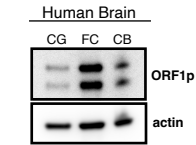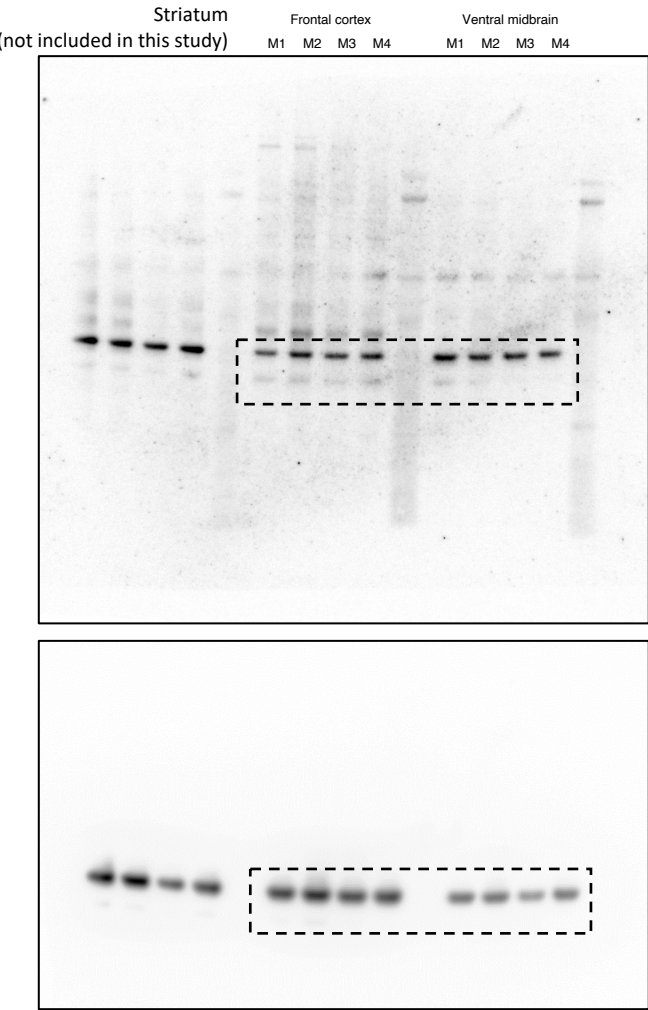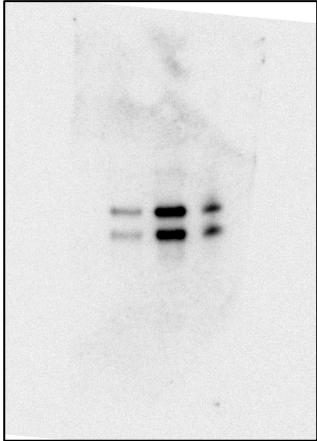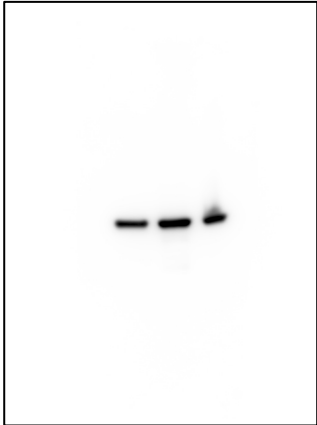

Supplement: Figure 1—source data 1. [file elife-100687-fig1-data1.zip › Figure 1 - source data 1 (pdf) /Figure 1 - source data.pdf]

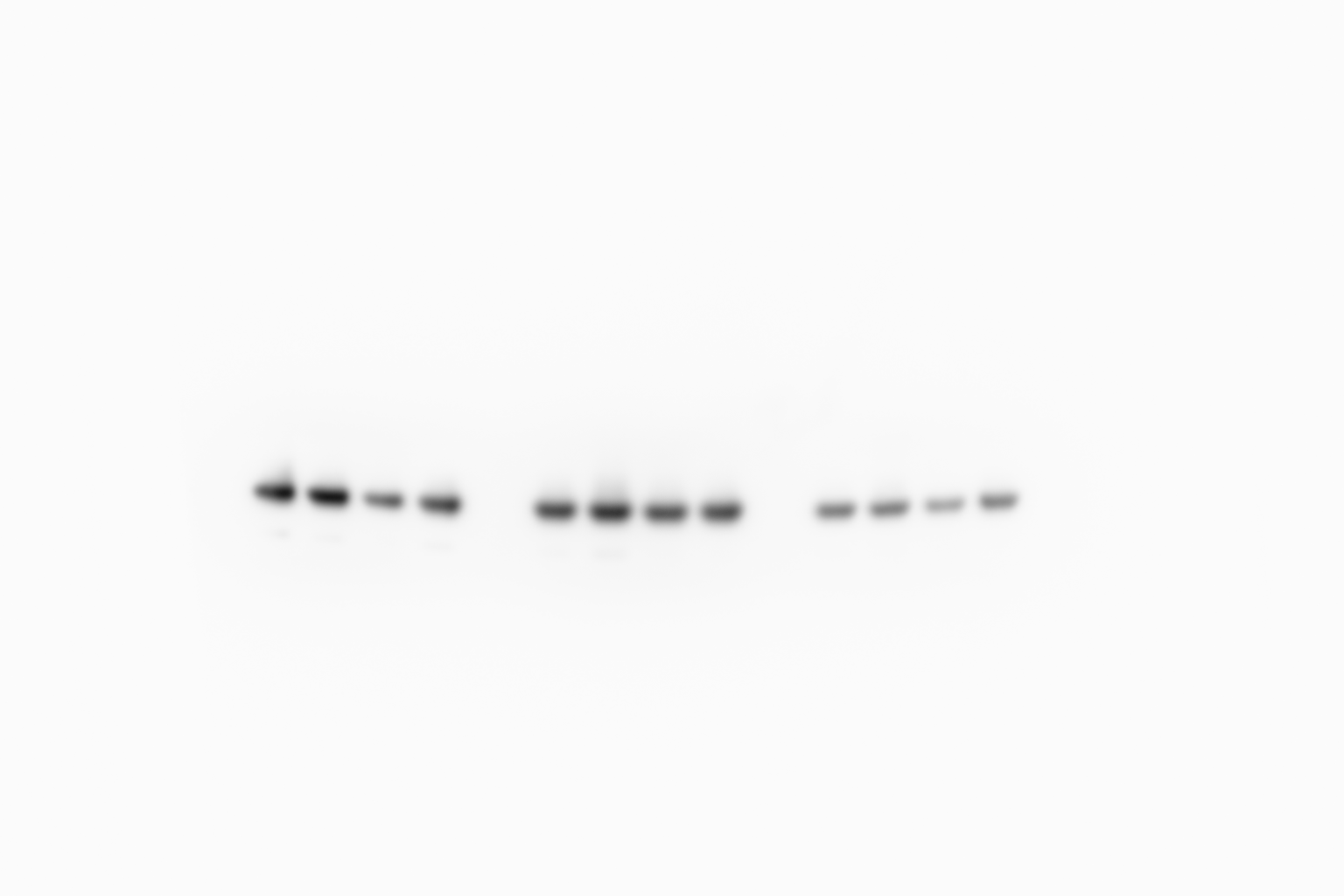

Supplement: Figure 1—source data 2. [file elife-100687-fig1-data2.zip › Figure 1 - source data 2 (raw)/Figure 1 - source data_corresponding WB actin four mice FC and vMB .tif]

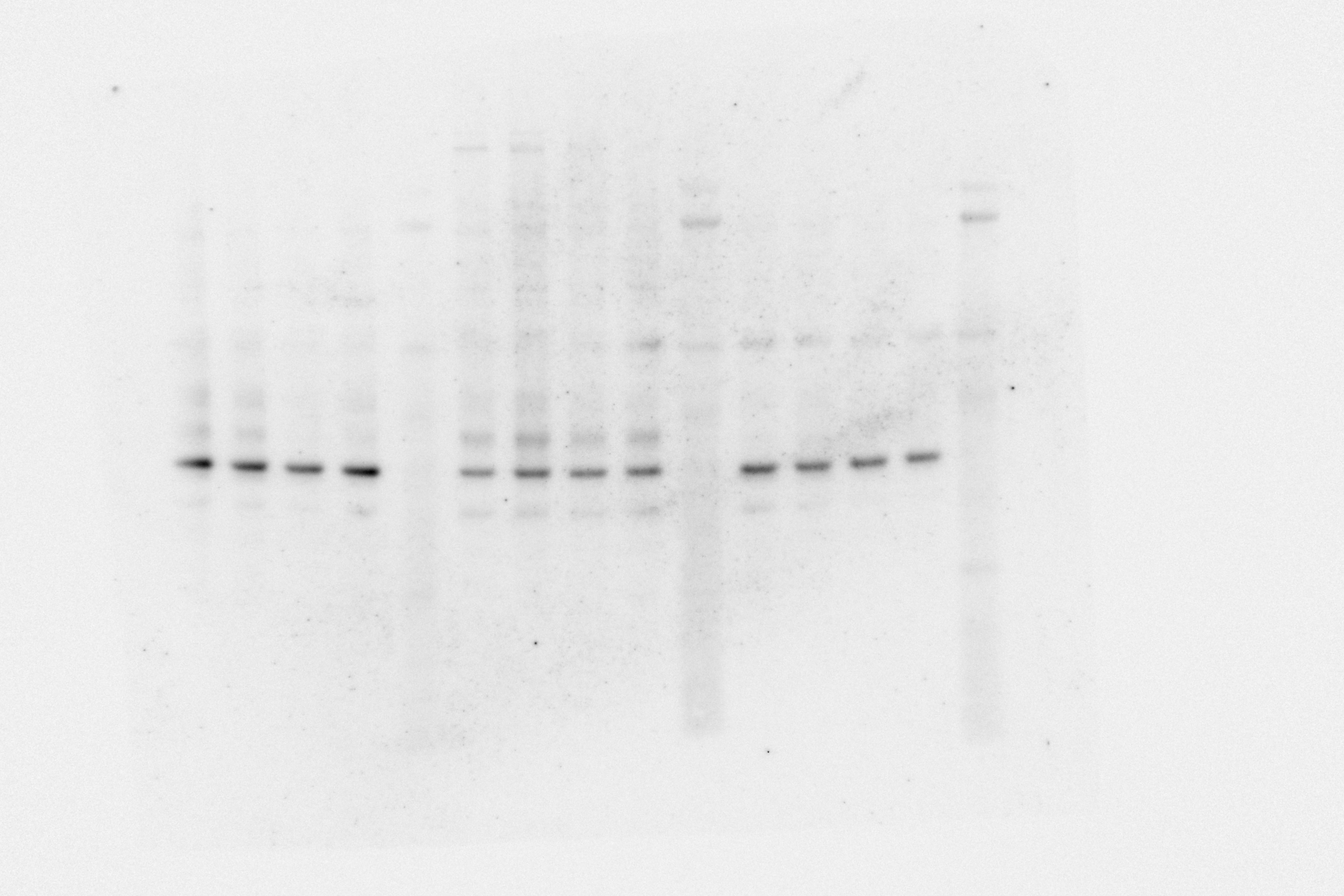

Supplement: Figure 1—source data 2. [file elife-100687-fig1-data2.zip › Figure 1 - source data 2 (raw)/Figure 1 - source data_WB ORF1p four mice FC and vMB.tif]

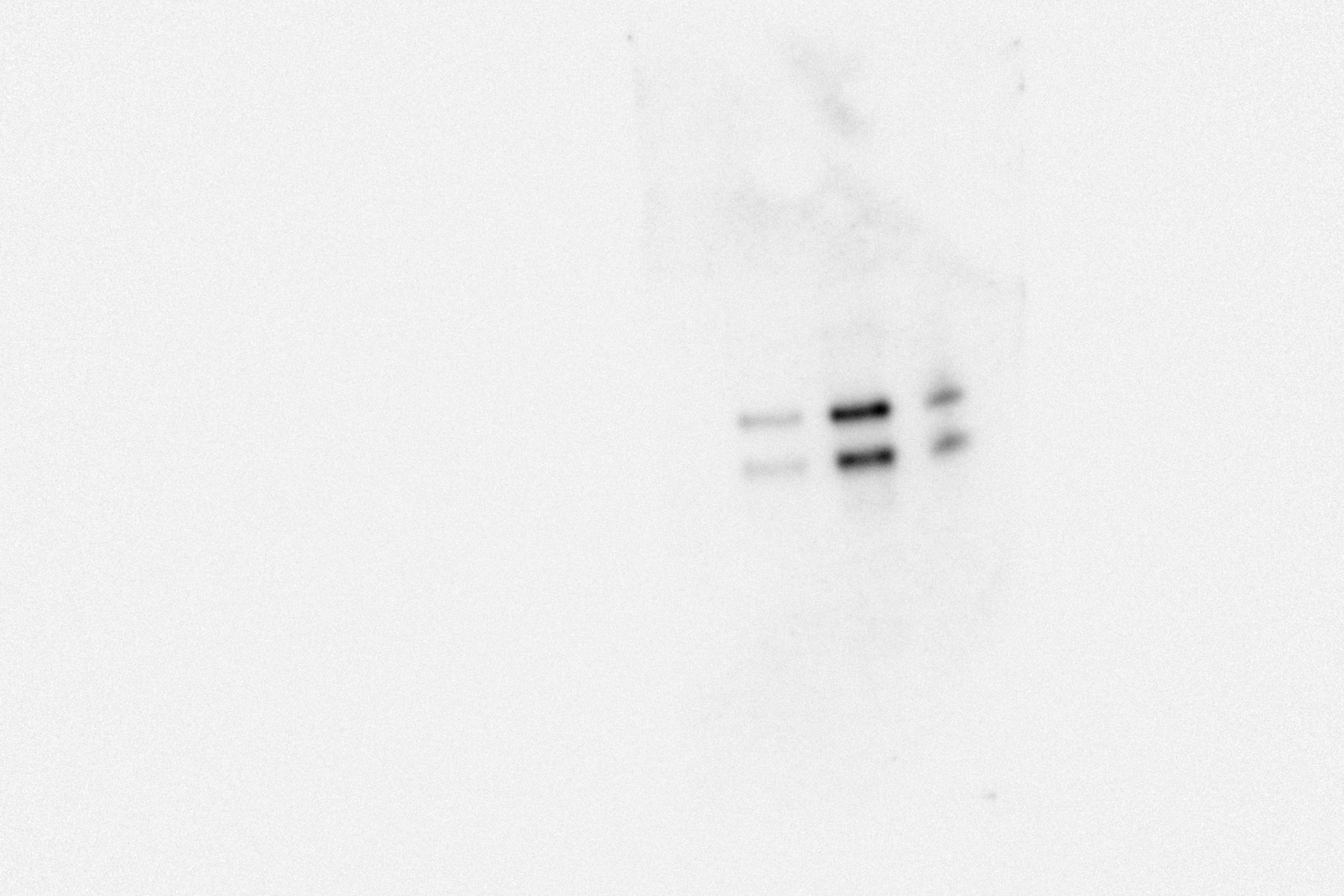

Supplement: Figure 1—source data 2. [file elife-100687-fig1-data2.zip › Figure 1 - source data 2 (raw)/Figure 1 - source data_WB ORF1p three brain regions human post-mortem.tif]

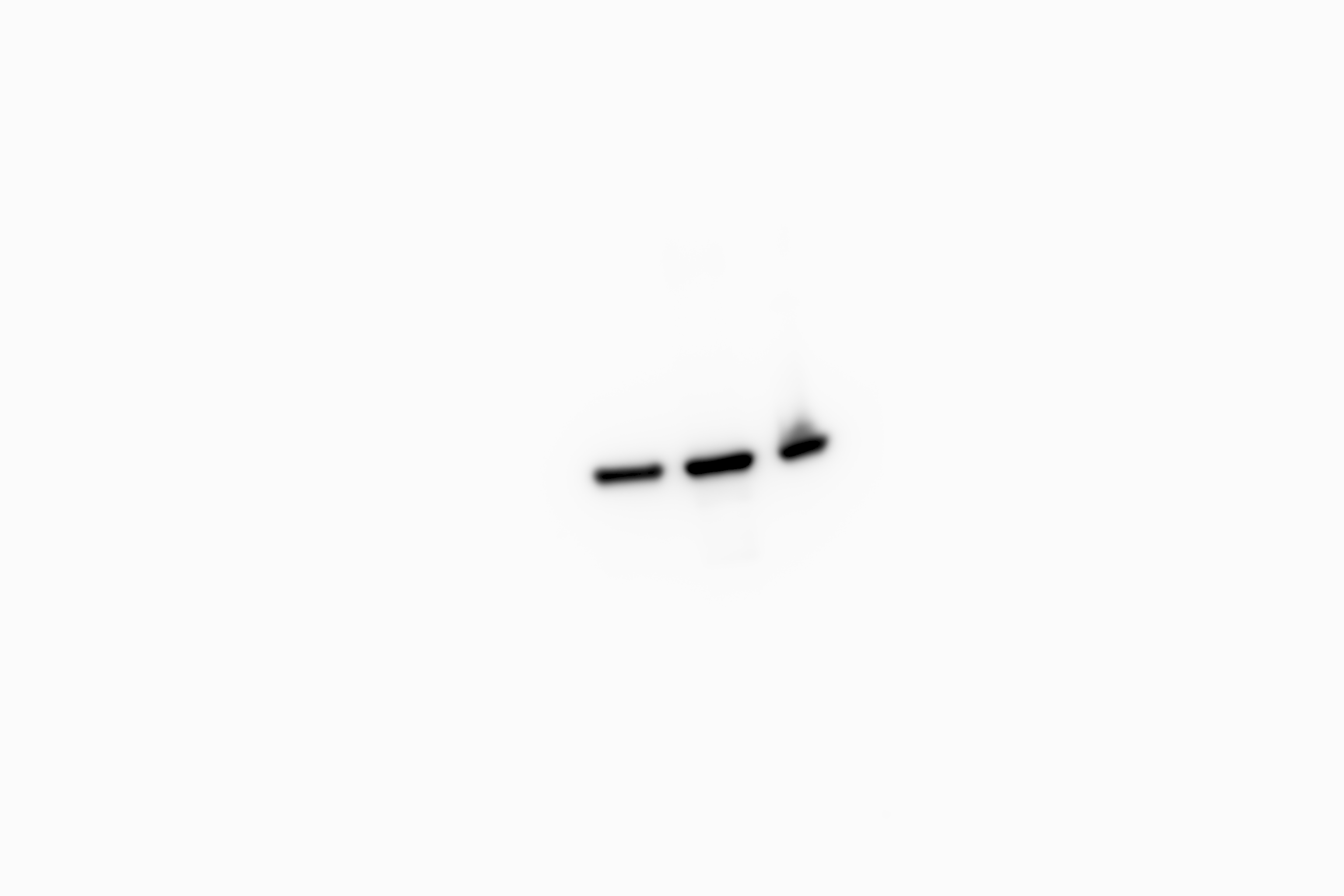

Supplement: Figure 1—source data 2. [file elife-100687-fig1-data2.zip › Figure 1 - source data 2 (raw)/Figure 1 - source data_corresponding WB three regions human post-mortem.tif]

Figure 1- figure supplement 1 –source data

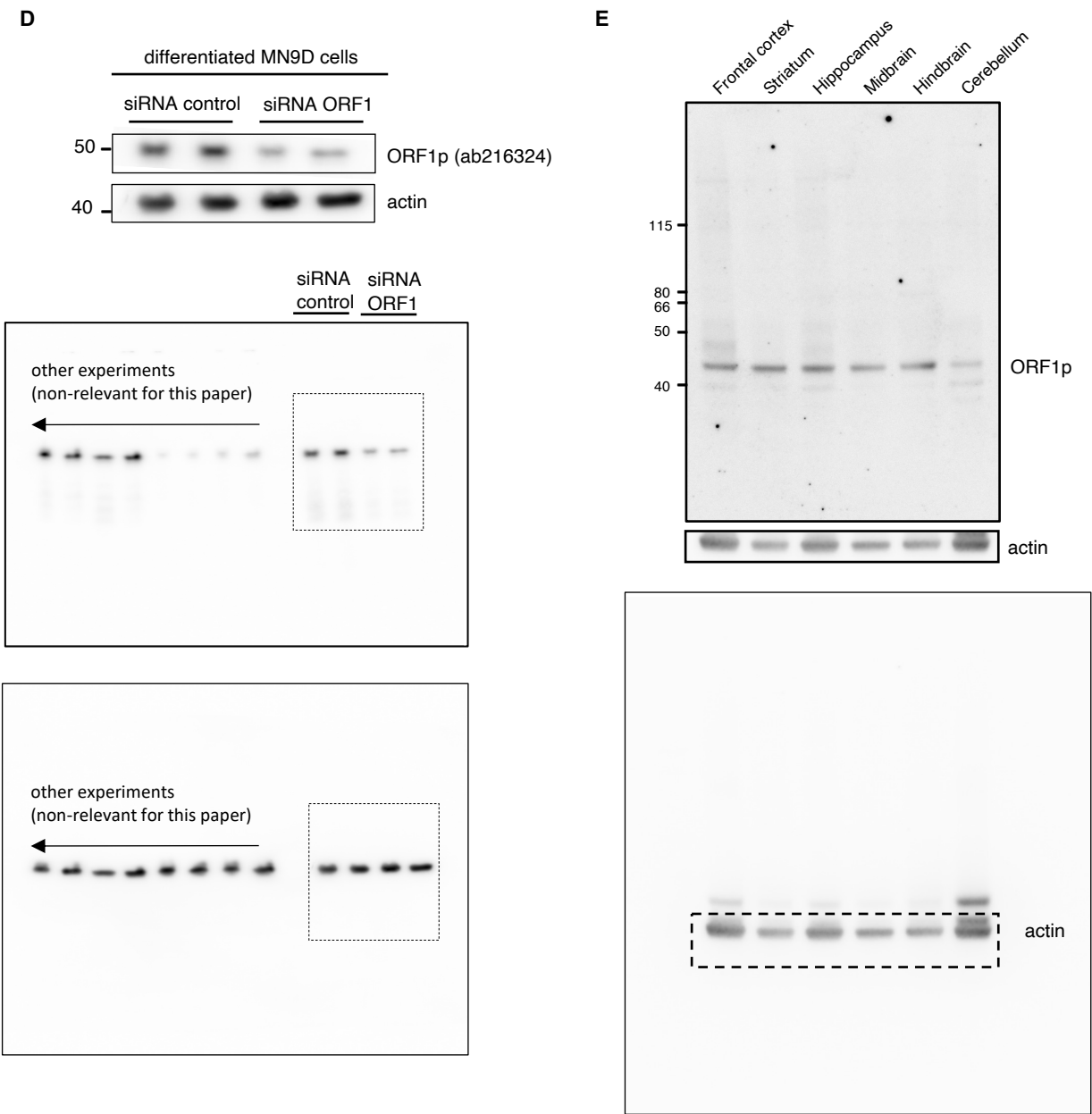

Supplement: Figure 1—figure supplement 1—source data 1. [file elife-100687-fig1-figsupp1-data1.zip › Figure 1 - figure supplement 1 - source data 1 (pdf)/Figure 1 - figure supplement 1 - source data .pdf]

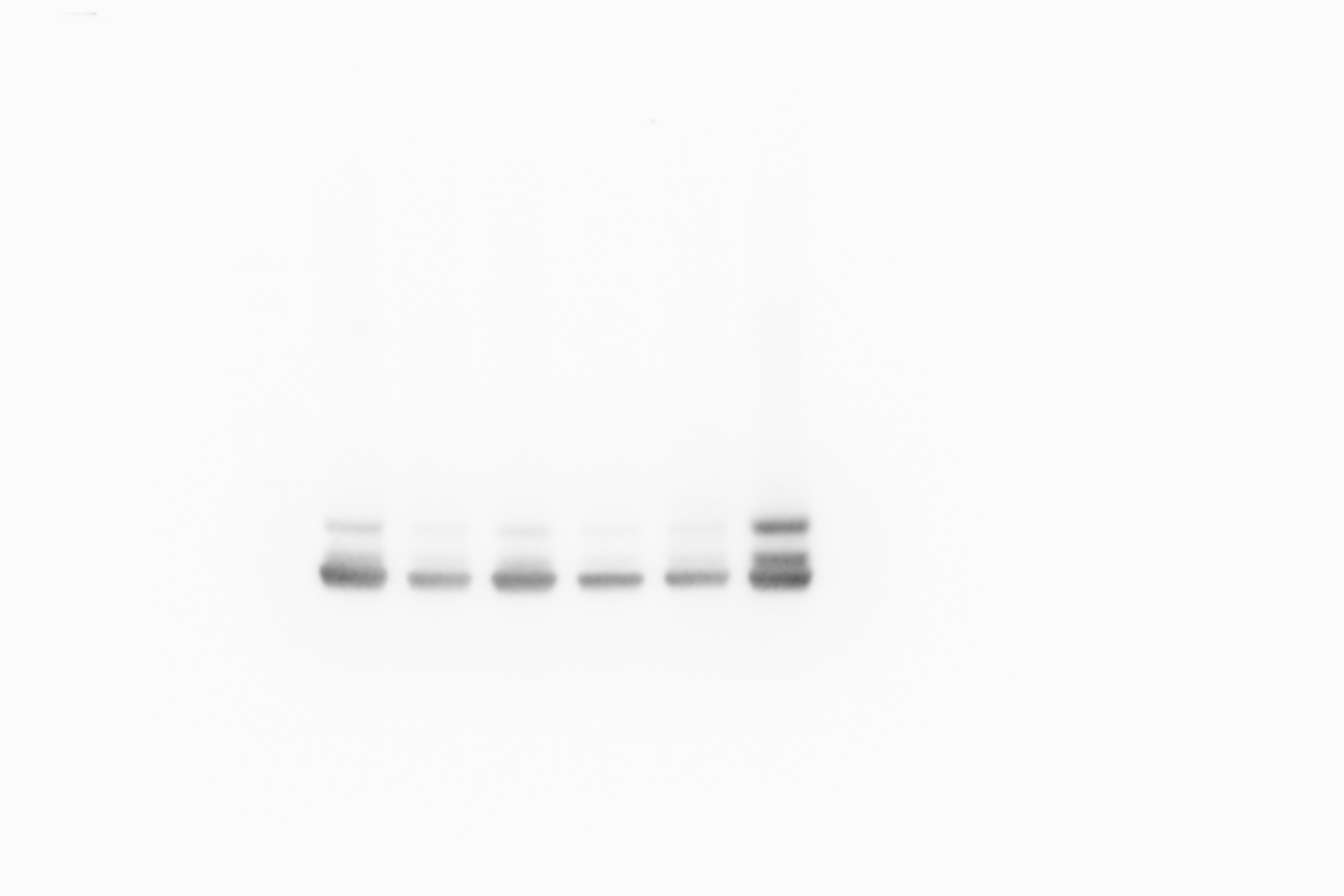

Supplement: Figure 1—figure supplement 1—source data 2. [file elife-100687-fig1-figsupp1-data2.zip › Figure 1 - figure supplement 1 - source data 2 (raw) /Figure 1 - figure supplement 1 - source data_corresponding WB actin 6 brain regions human post-mortem.tif]

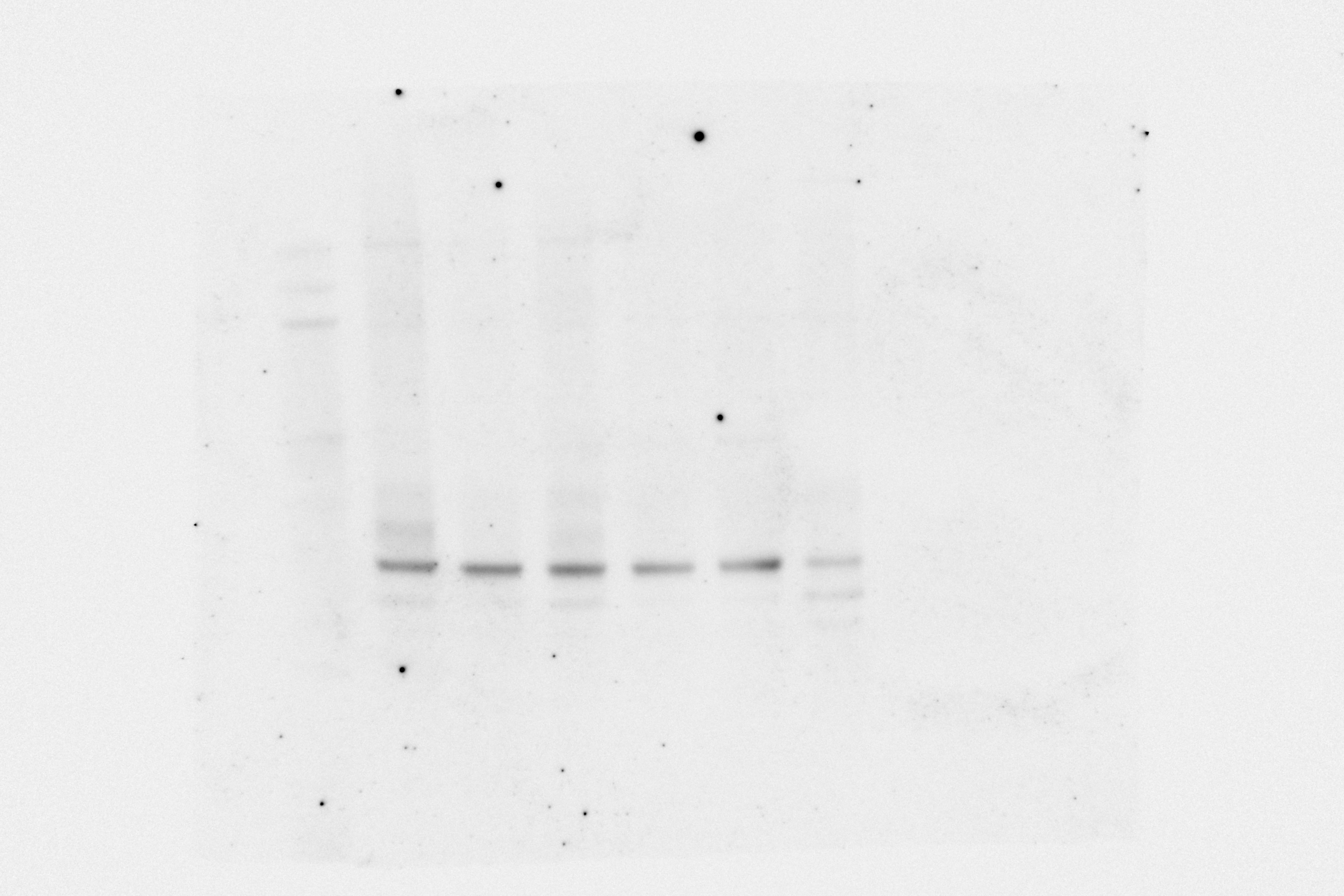

Supplement: Figure 1—figure supplement 1—source data 2. [file elife-100687-fig1-figsupp1-data2.zip › Figure 1 - figure supplement 1 - source data 2 (raw) /Figure 1 - figure supplement 1 - source data_WB ORF1p six brain regions human post-mortem.tif]

Figure 1- figure supplement 2- source data 2

C

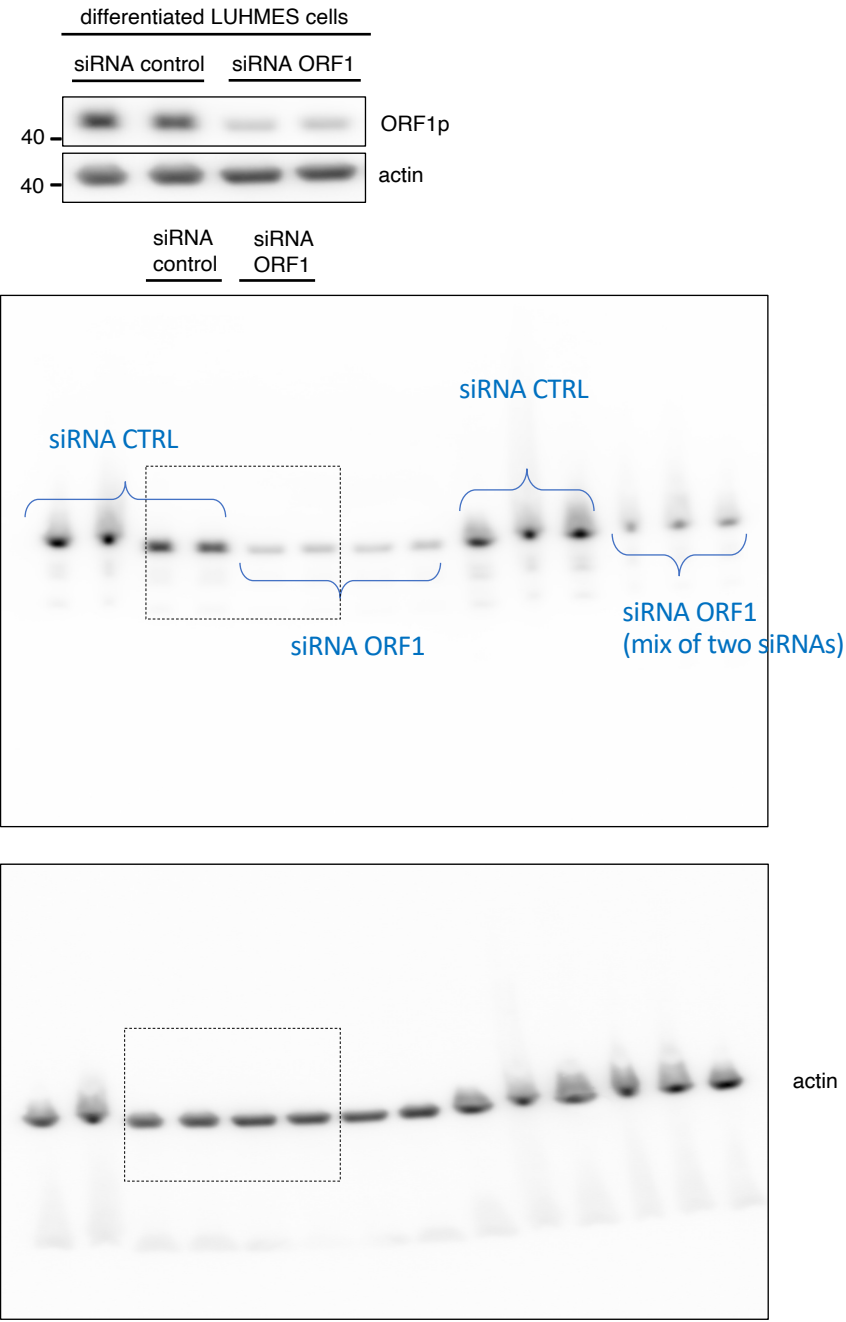

Supplement: Figure 1—figure supplement 2—source data 1. [file elife-100687-fig1-figsupp2-data1.zip › Figure 1 - figure supplement 2 - source data 1 (pdf)/Figure 1 - figure supplement 2 - source data 2.pdf]

Figure 1- figure supplement 2- source data 1

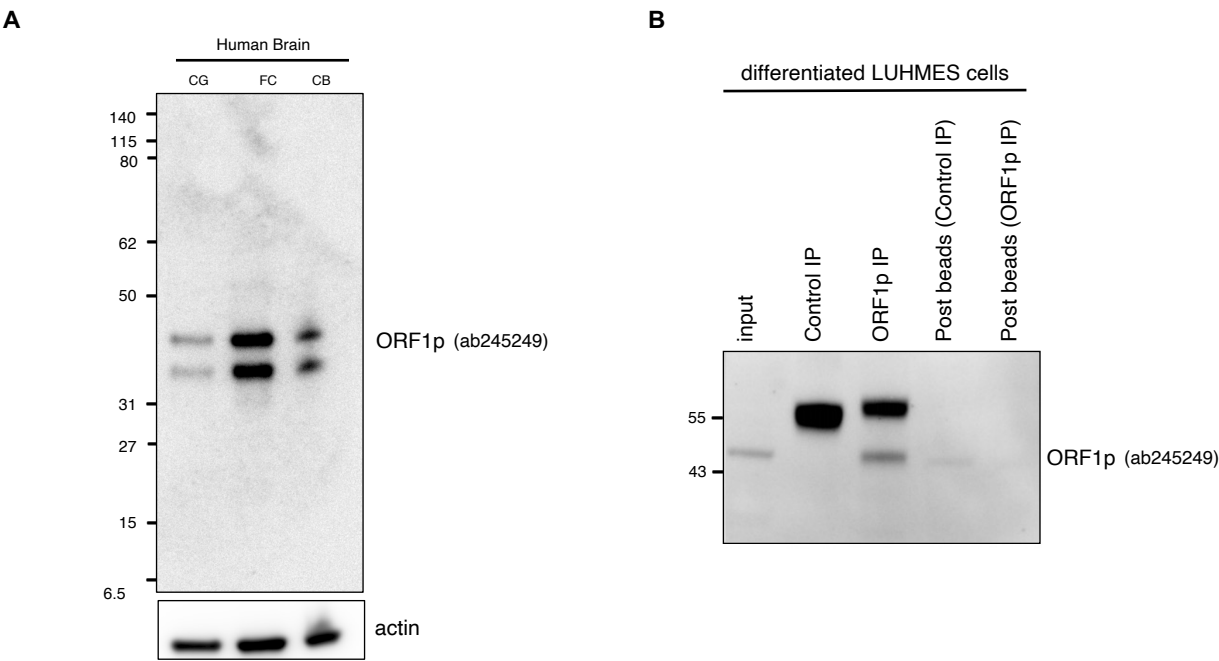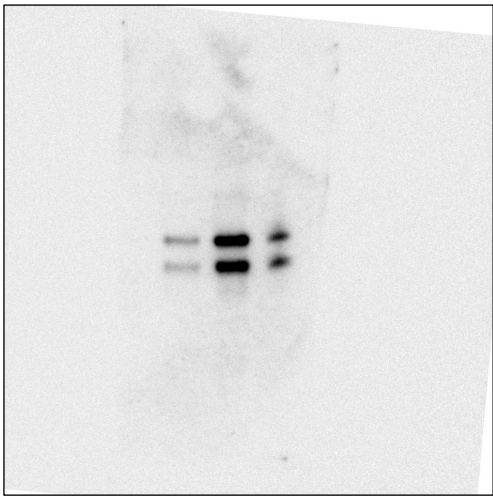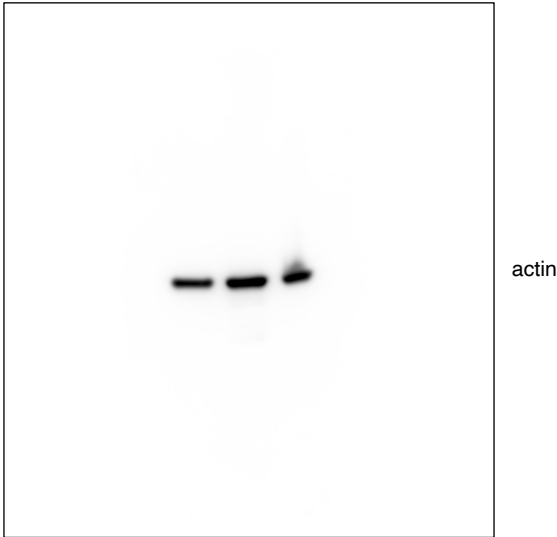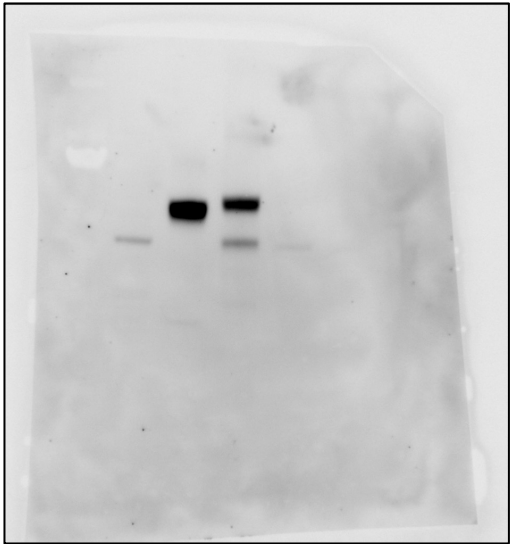

Supplement: Figure 1—figure supplement 2—source data 1. [file elife-100687-fig1-figsupp2-data1.zip › Figure 1 - figure supplement 2 - source data 1 (pdf)/Figure 1 - figure supplement 2 - source data 1.pdf]

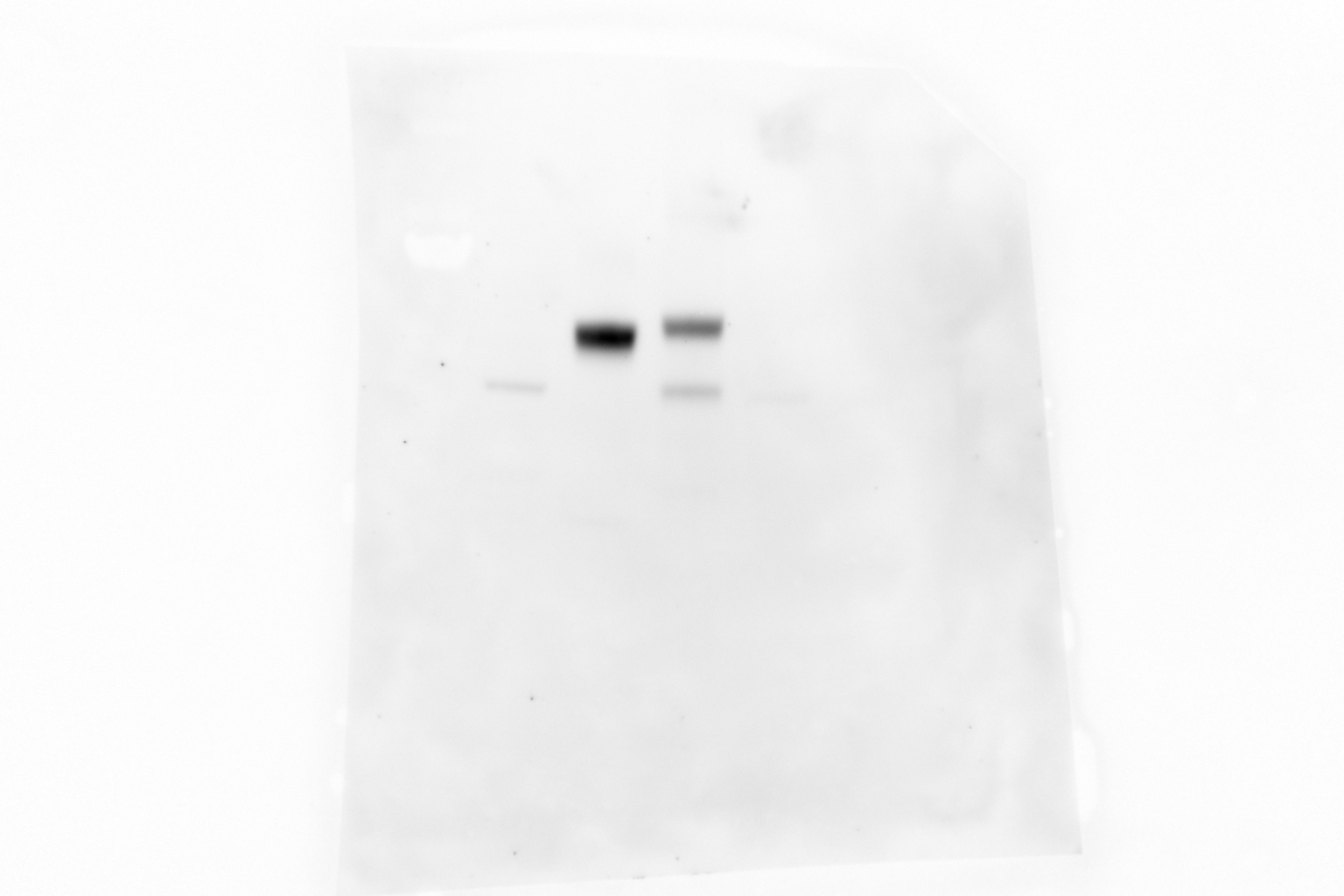

Supplement: Figure 1—figure supplement 2—source data 2. [file elife-100687-fig1-figsupp2-data2.zip › Figure 1 - figure supplement 2 - source data 2 (raw)/Figure 1 - figure supplement 2 - source data_IP ORF1p WB ORF1p LUHMES.tif]

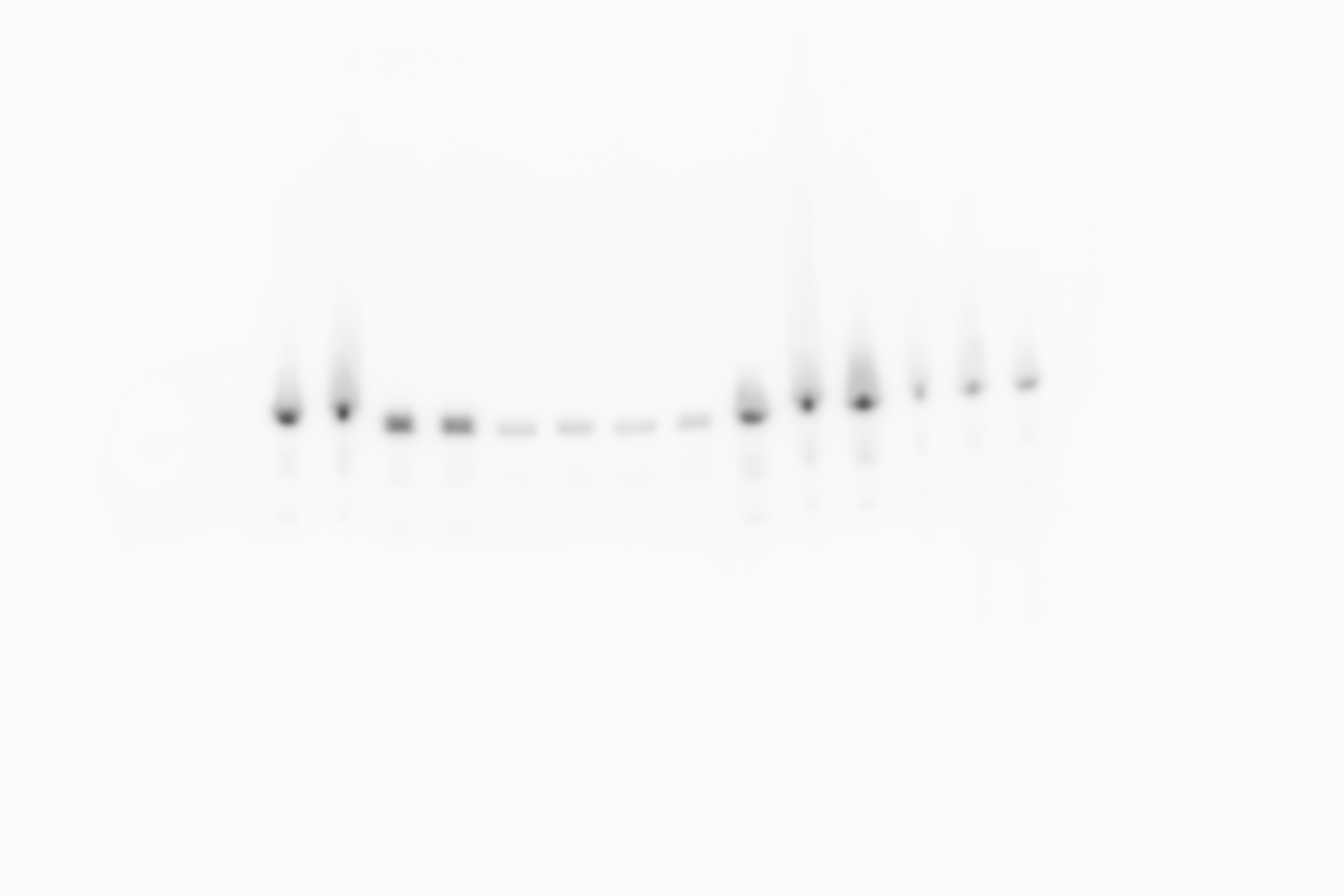

Supplement: Figure 1—figure supplement 2—source data 2. [file elife-100687-fig1-figsupp2-data2.zip › Figure 1 - figure supplement 2 - source data 2 (raw)/Figure 1 - figure supplement 2 - source data_WB ORF1p siRNA LUHMES .tif]

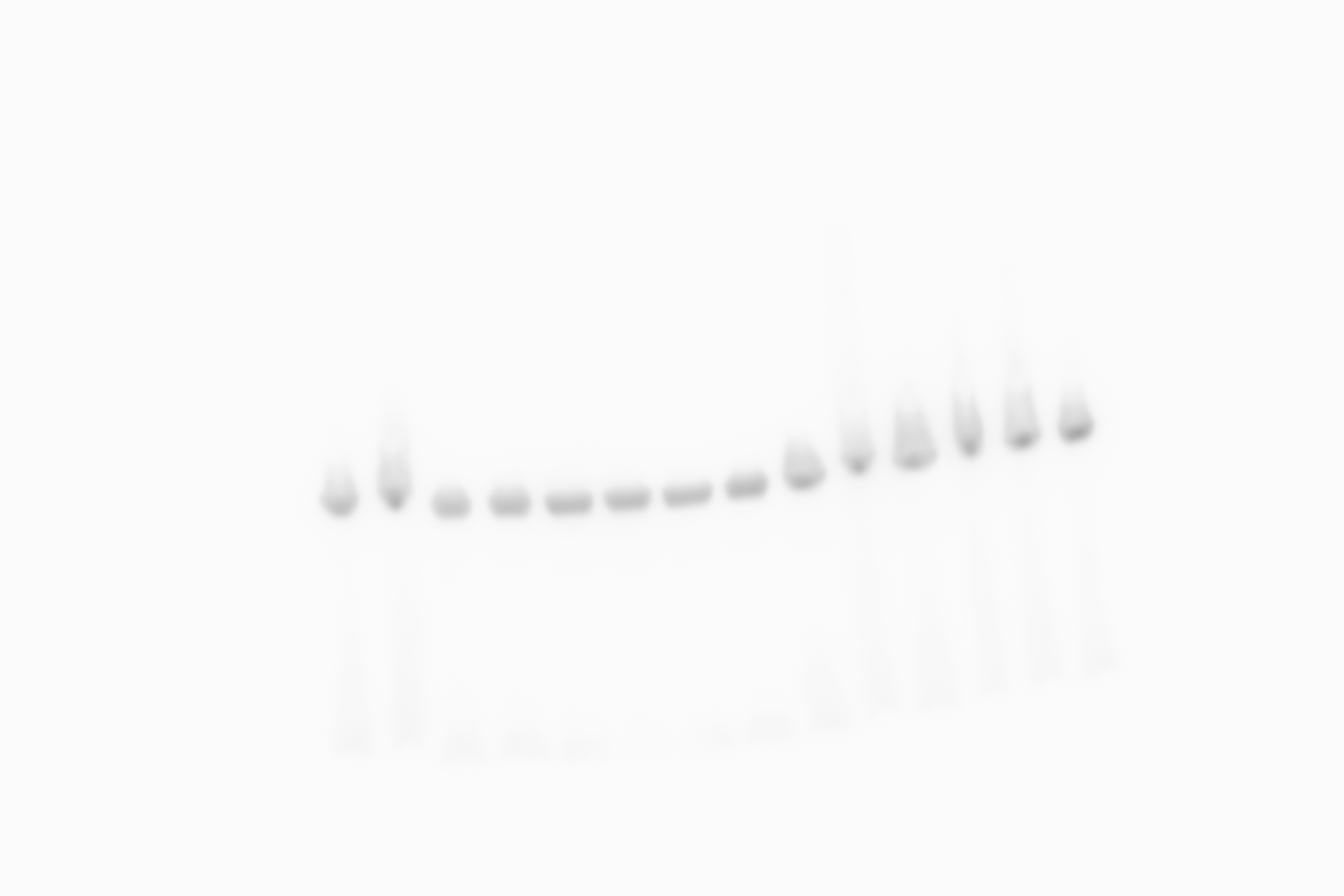

Supplement: Figure 1—figure supplement 2—source data 2. [file elife-100687-fig1-figsupp2-data2.zip › Figure 1 - figure supplement 2 - source data 2 (raw)/Figure 1 - figure supplement 2 - source data_corresponding WB actin siRNA LUHMES.tif]

Figure 2- Source data 1

G

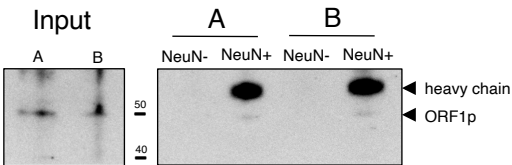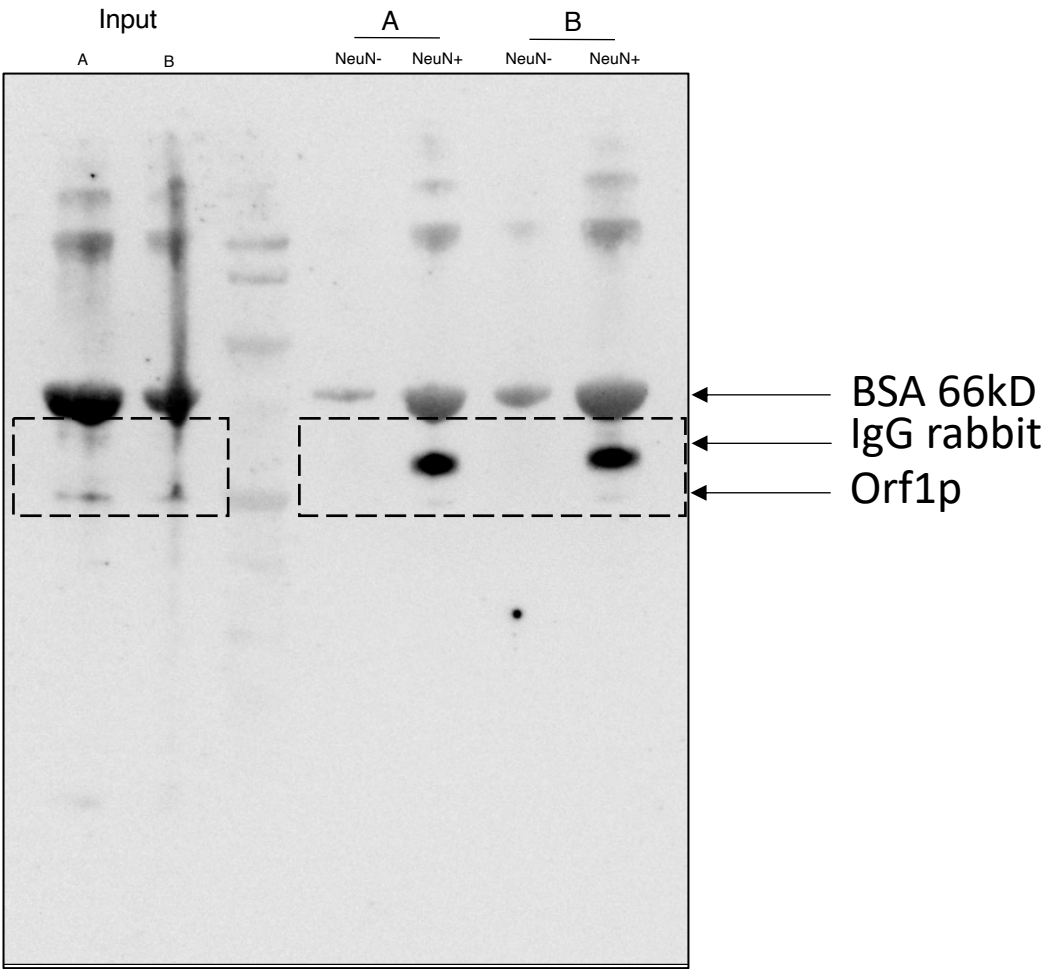

Supplement: Figure 2—source data 1. [file elife-100687-fig2-data1.zip › Figure 2 - source data 1 (pdf)/Figure 2 - source data.pdf]

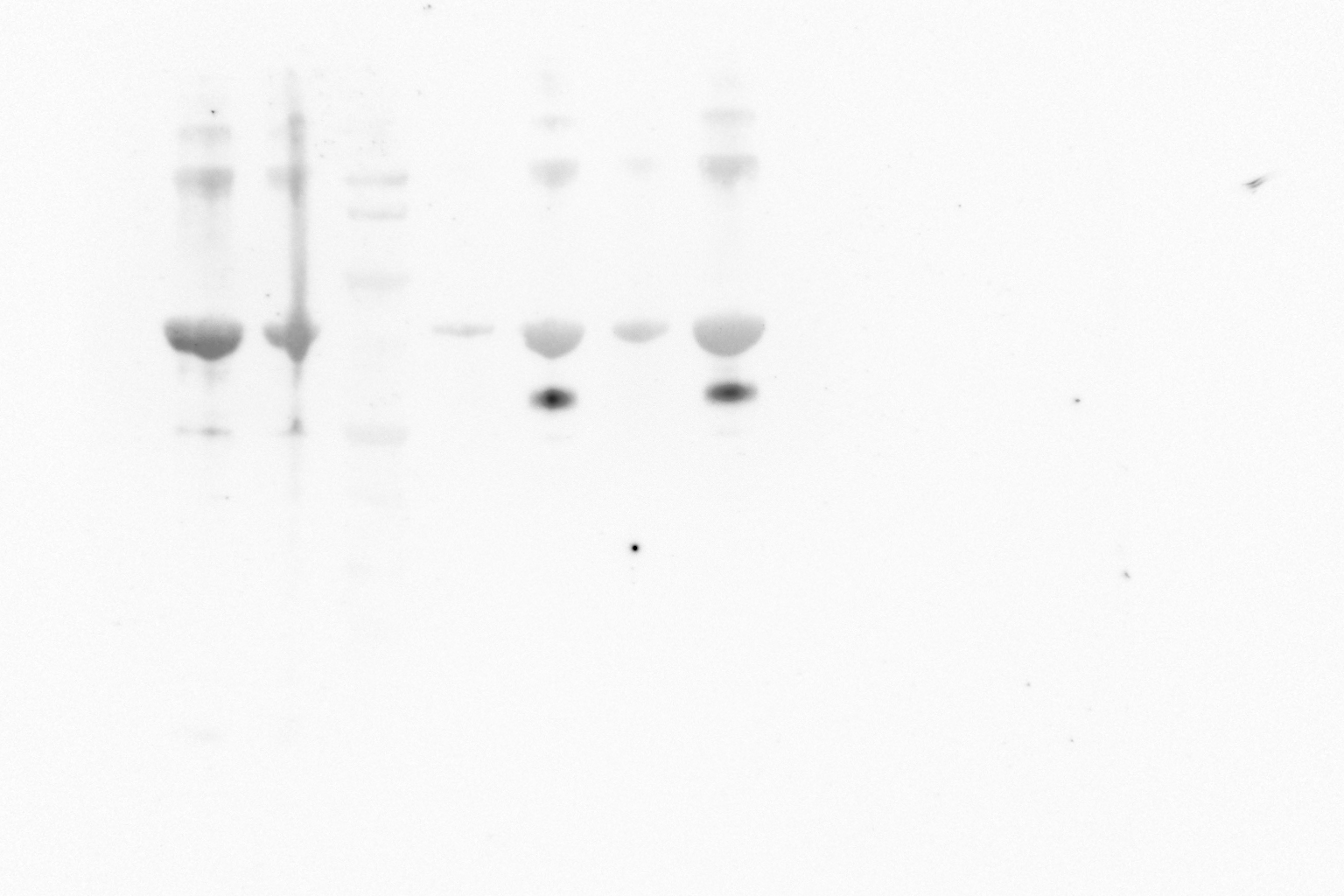

Supplement: Figure 2—source data 2. [file elife-100687-fig2-data2.zip › Figure 2 - source data 2 (raw)/Figure 2 - source data_WB ORF1p FACS NeuN.tif]

Figure 6 -Source data 1

A

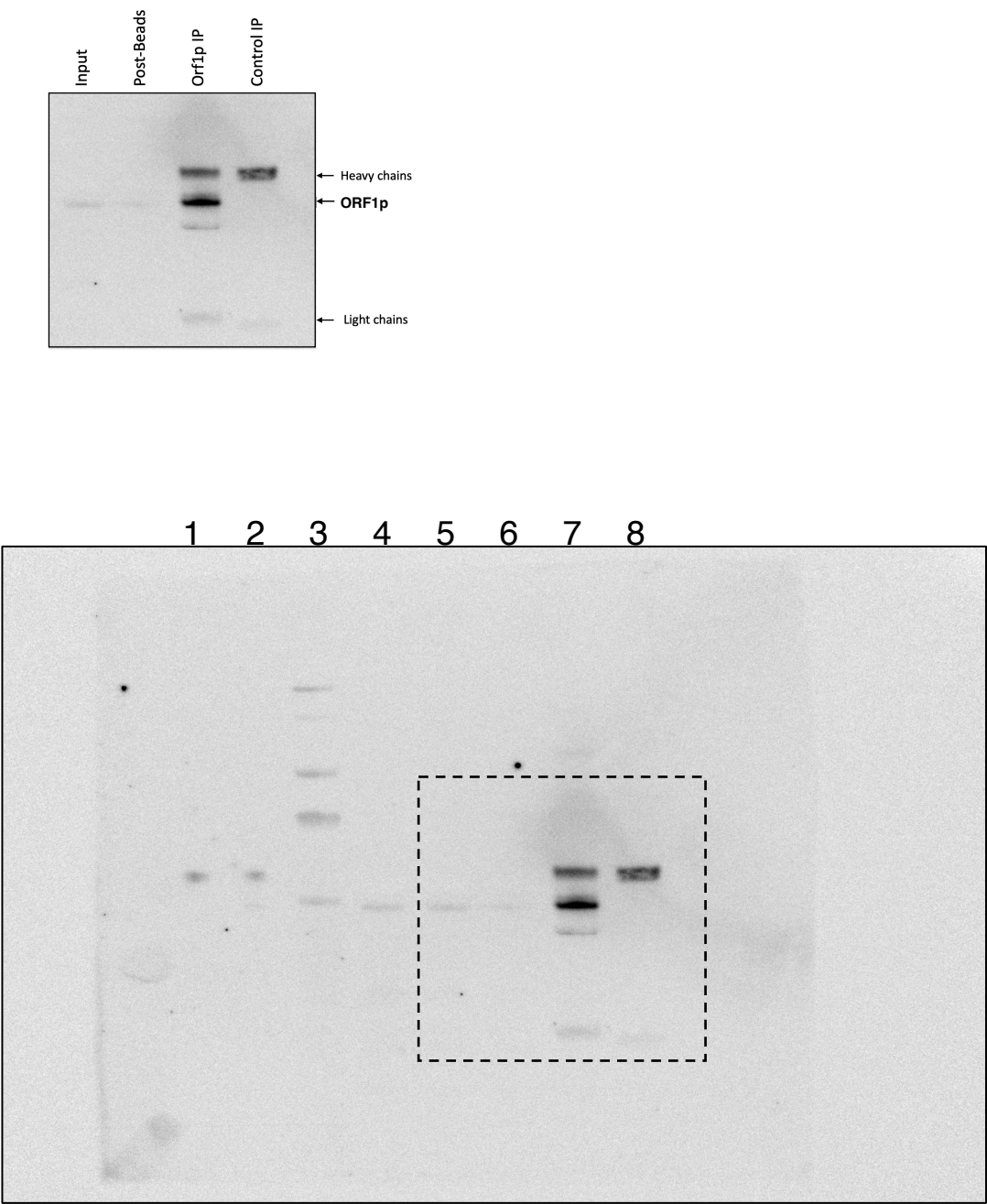

Supplement: Figure 6—source data 1. [file elife-100687-fig6-data1.zip › Figure 6 - source data 1 (pdf)/Figure 6 - source data 1.pdf]

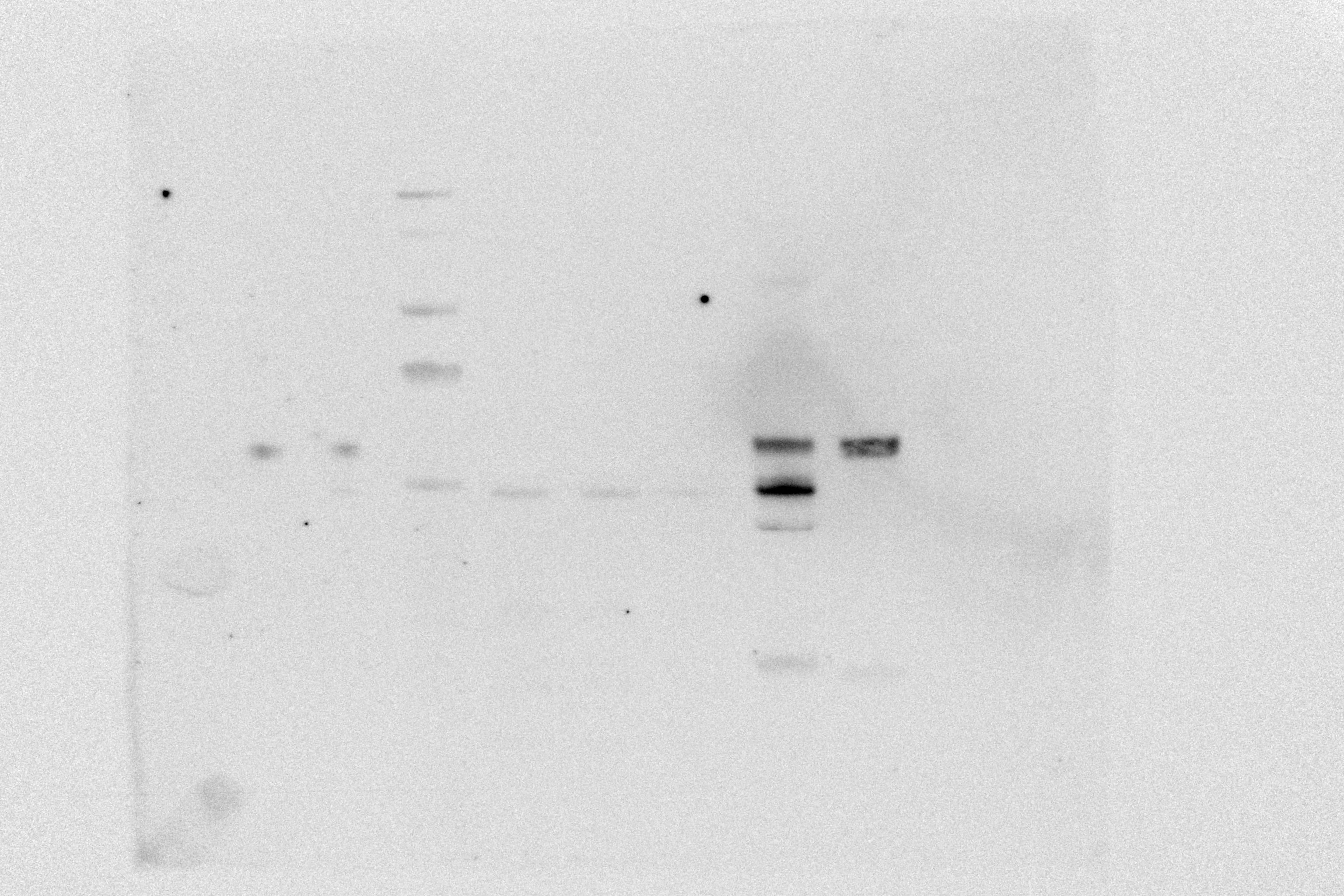

Supplement: Figure 6—source data 2. [file elife-100687-fig6-data2.zip › Figure 6 - source data 2.tif]
