# Supplementary material for: Steady-state neuron-predominant LINE-1 encoded ORF1p protein and LINE-1 RNA increase with aging in the mouse and human brain
Source: eLife. 2025 Sep 25;13:RP100687. doi: 10.7554/eLife.100687 (PMC12463392; doi:10.7554/eLife.100687)
Supplement: Supplementary file 3. [file elife-100687-supp3.xlsx]

PANTHER GO-Slim Biological Process	#	#	expected
RNA decapping	9	4	.17
cGMP-mediated signaling	11	4	.21
microtubule depolymerization	20	4	.39
adherens junction organization	26	5	.50
negative regulation of cytoskeleton organization	47	8	.91
negative regulation of supramolecular fiber organization	45	7	.87
nuclear-transcribed mRNA catabolic process	55	7	1.06
negative regulation of organelle organization	70	8	1.35
mRNA catabolic process	91	10	1.75
RNA catabolic process	108	10	2.08
negative regulation of cellular component organization	101	9	1.95
regulation of supramolecular fiber organization	93	8	1.79
regulation of translation	99	8	1.91
negative regulation of gene expression	157	12	3.03
posttranscriptional regulation of gene expression	136	10	2.62
cell junction organization	163	11	3.14
nucleobase-containing compound catabolic process	149	10	2.87
organic cyclic compound catabolic process	183	12	3.53
aromatic compound catabolic process	170	11	3.28
neuron projection development	217	14	4.18
cell morphogenesis	237	15	4.57
actin filament organization	198	12	3.82
neuron development	235	14	4.53
actin cytoskeleton organization	290	17	5.59
actin filament-based process	299	17	5.76
supramolecular fiber organization	320	18	6.17
synaptic signaling	268	14	5.17
regulation of cellular component organization	369	19	7.11
cytoskeleton organization	713	35	13.74
cell-cell signaling	419	20	8.08
microtubule-based process	481	22	9.27
nervous system development	483	22	9.31
plasma membrane bounded cell projection organization	420	19	8.10
cell projection organization	425	19	8.19
intracellular signal transduction	835	34	16.09
movement of cell or subcellular component	650	26	12.53
regulation of cell communication	790	30	15.23
regulation of signaling	791	30	15.25
cellular component assembly	867	32	16.71
cellular component organization	2624	82	50.58
organelle organization	1998	61	38.51
cellular component organization or biogenesis	2745	82	52.91
cellular process	10403	235	200.52

old Enrichmer	+/-	raw P value	FDR
23.06	+	7.87E-05	1.94E-02
18.87	+	1.46E-04	1.70E-02
10.38	+	9.93E-04	4.90E-02
9.98	+	2.68E-04	2.58E-02
8.83	+	8.56E-06	6.34E-03
8.07	+	5.29E-05	1.68E-02
6.60	+	1.65E-04	1.83E-02
5.93	+	1.13E-04	1.57E-02
5.70	+	2.18E-05	9.70E-03
4.80	+	8.34E-05	1.68E-02
4.62	+	2.45E-04	2.47E-02
4.46	+	6.68E-04	4.01E-02
4.19	+	9.77E-04	5.05E-02
3.97	+	9.42E-05	1.49E-02
3.81	+	4.75E-04	3.29E-02
3.50	+	4.97E-04	3.34E-02
3.48	+	9.21E-04	4.99E-02
3.40	+	3.57E-04	2.83E-02
3.36	+	6.91E-04	4.04E-02
3.35	+	1.39E-04	1.71E-02
3.28	+	9.90E-05	1.46E-02
3.14	+	6.93E-04	3.94E-02
3.09	+	3.01E-04	2.67E-02
3.04	+	8.47E-05	1.57E-02
2.95	+	1.20E-04	1.57E-02
2.92	+	8.69E-05	1.48E-02
2.71	+	1.03E-03	4.85E-02
2.67	+	1.67E-04	1.76E-02
2.55	+	1.41E-06	3.14E-03
2.48	+	2.92E-04	2.70E-02
2.37	+	3.21E-04	2.74E-02
2.36	+	3.33E-04	2.74E-02
2.35	+	9.74E-04	5.15E-02
2.32	+	1.06E-03	4.92E-02
2.11	+	6.37E-05	1.77E-02
2.08	+	7.54E-04	4.19E-02
1.97	+	5.80E-04	3.78E-02
1.97	+	5.88E-04	3.73E-02
1.91	+	6.32E-04	3.90E-02
1.62	+	1.53E-05	8.51E-03
1.58	+	4.58E-04	3.39E-02
1.55	+	8.15E-05	1.81E-02
1.17	+	9.86E-04	4.97E-02
