# Supplementary material for: Steady-state neuron-predominant LINE-1 encoded ORF1p protein and LINE-1 RNA increase with aging in the mouse and human brain
Source: eLife. 2025 Sep 25;13:RP100687. doi: 10.7554/eLife.100687 (PMC12463392; doi:10.7554/eLife.100687)
Supplement: Supplementary file 4. [file elife-100687-supp4.xlsx]

Gene ref	Gene name	Condition
P11260	L1RE1	known
Q6ZQ58	LARP1	known
Q64012	RALY	known
Q80ZM5	H1FX	known
Q8CJ67	STAU2	known
Q9JIK5	DDX21	known
F8VPP8	ZC3H7B	known
O70305	ATXN2	known
O88532	ZFR	known
P05784	KRT18	known
P29788	VTN	known
P97473	TARBP2	known
Q00623	APOA1	known
Q01405	SEC23A	known
Q3U564	DCP1B	known
Q3UJB9	EDC4	known
Q64337	SQSTM1	known
Q70FJ1	AKAP9	known
Q80TV8	CLASP1	known
Q8BH15	CNOT10	known
Q8BQZ5	CPSF4	known
Q8C080	SNX16	known
Q8C170	MYO9A	known
Q8K224	NAT10	known
Q8K2D3	EDC3	known
Q8VEF1	GRAMD1A	known
Q8VEH2	CIZ1	known
Q8VHK9	DHX36	known
Q91WJ7	SPATS2L	known
Q923J1	TRPM7	known
Q99MR1	GIGYF1	known
Q99NB9	SF3B1	known
Q99PU8	DHX30	known
Q9CWN7	CNOT11	known
Q9D6Z1	NOP56	known
Q9WTX2	PRKRA	known
Q9WVR4	FXR2	known
Q9Z108	STAU1	known
A0A2I3BQ48	DGKH	not known

A0A571BF58	NEB	not known
A0A5F8MPP0	PLEKHG1	not known
A2A6T1	CDR2L	not known
A2AAX3	KLHL15	not known
A2AF47	DOCK11	not known
A2AHC3	CAMSAP1	not known
A2AI08	TPRN	not known
A2AJK6	CHD7	not known
A2ALK8	PTPN3	not known
A2ALS5	RAP1GAP	not known
A2APX8	SCN1A	not known
A2AQ25	SKT	not known
A2ASZ8	SLC25A25	not known
A2AV25	FIBCD1	not known
A2BH40	ARID1A	not known
B1AWC9	PDE4B	not known
B2RR83	YTHDC2	not known
D3YXK0	JAKMIP2	not known
D3YZG8	MTHFD2L	not known
E9PUQ8	DGKD	not known
E9PZP8	HERC1	not known
E9Q0S6	TNS1	not known
E9Q1M1	PDZD2	not known
E9Q236	ABCC4	not known
E9Q401	RYR2	not known
E9Q4N7	ARID1B	not known
E9Q6P5	TTC7B	not known
E9Q6R7	UTRN	not known
E9Q8I9	FRY	not known
E9Q9B3	SPRYD3	not known
E9Q9B7	KIDINS220	not known
E9Q9K8	AKAP6	not known
E9Q9R9	DLG5	not known
F2Z3U3	RAPH1	not known
F7BAB2	TMEM132B	not known
F8VPN4	AGL	not known
F8VQB6	MYO1	not known
F8VQK3	GUCY1A2	not known
G3X9I7	ZC3H12B	not known
J9S314	TRPM3	not known
O08576	RUNDC3A	not known
O08672	KIFC2	not known
O09161	CASQ2	not known
O35066	KIF3C	not known
O35099	MAP3K5	not known

O35231	KIFC3	not known
O35643	AP1B1	not known
O35874	SLC1A4	not known
O54781	SRPK2	not known
O54828	RGS9	not known
O54865	GUCY1B3	not known
O70161	PIP5K1C	not known
O70378	EMC8	not known
O70405	ULK1	not known
O88196	TTC3	not known
O88384	VTI1B	not known
O88441	MTX2	not known
O88951	LIN7B	not known
O89084	PDE4A	not known
P08775	POLR2A	not known
P0C673	IGSF11	not known
P11531	DMD	not known
P11679	KRT8	not known
P16283	SLC4A3	not known
P28028	BRAF	not known
P28653	BGN	not known
P28654	DCN	not known
P28741	KIF3A	not known
P35585	AP1M1	not known
P37040	POR	not known
P39087	GRIK2	not known
P43024	COX6A1	not known
P45481	CREBBP	not known
P48725	PCNT	not known
P52332	JAK1	not known
P54818	GALC	not known
P55284	CDH5	not known
P58390	KCNN2	not known
P58391	KCNN3	not known
P59759	MKL2	not known
P60191	RIMS4	not known
P62046	LRCH1	not known
P62880	GNB2	not known
P70188	KIFAP3	not known
P70248	MYO1F	not known
P70257	NFIX	not known
P70271	PDLIM4	not known
P70297	STAM	not known
P80560	PTPRN2	not known
P97291	CDH8	not known

P97412	LYST	not known
P97467	PAM	not known
P97760	POLR2C	not known
P98084	APBA2	not known
Q03137	EPHA4	not known
Q03173	ENAH	not known
Q03717	KCNB	not known
Q04735	CDK16	not known
Q04859	MAK	not known
Q05860	FMN1	not known
Q0VAV2	EXPH	not known
Q0VGU4	VGF	not known
Q0VGY8	TANC1	not known
Q14BI2	GRM2	not known
Q2KN98	SPECC1L	not known
Q2PZL6	FAT4	not known
Q3TDQ1	STT3B	not known
Q3TE80	2410002F23RIK	not known
Q3TL44	NLRX1	not known
Q3TLI0	TRAPPC10	not known
Q3TRM4	PNPLA6	not known
Q3TRR0	MAP9	not known
Q3TVI8	PBXIP1	not known
Q3TY86	AIFM3	not known
Q3U0M1	TRAPPC9	not known
Q3U5C7	PRICKLE1	not known
Q3UE31	MTUS2	not known
Q3UHC7	DAB2IP	not known
Q3UHD1	BAI1	not known
Q3UHF7	HIVEP2	not known
Q3UHH1	ZSWIM8	not known
Q3UHI0	CCSER2	not known
Q3UHU5	SOGA2	not known
Q3ULB5	PAK6	not known
Q3ULW6	CCDC33	not known
Q3UMT1	PPP1R12C	not known
Q3UNH4	GPRIN1	not known
Q3URD3	SLMAP	not known
Q3USH1	FAM196A	not known
Q3UTQ8	CDKL5	not known
Q3UTZ3	MAP11	not known
Q3UUF8	ANKRD34B	not known
Q3UV70	PDP1	not known
Q3UVC0	KSR2	not known
Q3UVX5	GRM5	not known

Q3V1U8	ELMOD1	not known
Q499E5	STOX2	not known
Q4QQM5	FAM73A	not known
Q4U2R1	HERC2	not known
Q4VAC9	PLEKHG3	not known
Q505D1	ANKRD28	not known
Q571I4	SGK223	not known
Q5DTY9	KCTD16	not known
Q5DU14	MYO10	not known
Q5HZI2	C2CD4C	not known
Q5NBX1	COBL	not known
Q5RJH3	CDH12	not known
Q5RJH6	SMG7	not known
Q5SUA5	MYO1G	not known
Q5SW75	SSH2	not known
Q60737	CSNK2A1	not known
Q60770	STXBP3	not known
Q60803	TRAF3	not known
Q60841	RELN	not known
Q60900	ELAVL3	not known
Q60996	PPP2R5C	not known
Q61033	TMPO	not known
Q61315	APC	not known
Q61334	BCAP29	not known
Q61464	ZNF638	not known
Q61771	KIF3B	not known
Q62095	DDX3Y	not known
Q62120	JAK2	not known
Q62407	SPEG	not known
Q64096	MCF2L	not known
Q640Q5	PAN3	not known
Q641K5	NUAK1	not known
Q64727	VCL	not known
Q65CL1	CTNNA3	not known
Q67BT3	SLC13A5	not known
Q68FF0	KIAA1841	not known
Q69Z99	ZNF512	not known
Q69ZS8	KAZN	not known
Q6DFV5	HELZ	not known
Q6DIC0	SMARCA2	not known
Q6GQW0	BTBD11	not known
Q6IMP4	PANX2	not known
Q6NXI6	RPRD2	not known
Q6NXJ0	WWC2	not known
Q6P6I6	POLR2M	not known

Q6PAM0	PRKAB2	not known
Q6PCX9	TRIM37	not known
Q6PCZ4	MAGEE1	not known
Q6PD28	PPP2R5B	not known
Q6PDG5	SMARCC2	not known
Q6PEE2	CTIF	not known
Q6PEM6	GRAMD3	not known
Q6PHZ8	KCNIP4	not known
Q6RHR9	MAGI1	not known
Q6RI63	FAM120B	not known
Q6Y7W8	GIGYF2	not known
Q6ZPQ6	PITPNM2	not known
Q6ZPY2	SMG5	not known
Q6ZQ29	TAOK2	not known
Q70IV5	SYNM	not known
Q76I79	SSH1	not known
Q7TME0	PLPPR4	not known
Q7TNM2	TRIM46	not known
Q7TPV4	MYBBP1A	not known
Q7TQ95	LNP	not known
Q7TQF2	FBXO10	not known
Q7TT37	IKBKAP	not known
Q7TT50	CDC42BPB	not known
Q80TF3	PCDH19	not known
Q80TL0	PPM1E	not known
Q80TM6	R3HDM2	not known
Q80TM9	NISCH	not known
Q80TN7	NAV3	not known
Q80TT8	CUL9	not known
Q80TZ9	RERE	not known
Q80U04	PJA2	not known
Q80U19	DAAM2	not known
Q80U59	KIAA0232	not known
Q80UK0	SESTD1	not known
Q80V91	DTX3	not known
Q80W47	WIPI2	not known
Q80WG5	LRRC8A	not known
Q80X66	BTBD10	not known
Q80Y83	DIXDC1	not known
Q80YA3	DDHD1	not known
Q80YE7	DAPK1	not known
Q80YF9	ARHGAP33	not known
Q80YT7	PDE4DIP	not known
Q80ZJ7	SNX32	not known
Q80ZX0	SEC24B	not known

Q8BG89	ZNF365	not known
Q8BI72	CDKN2AIP	not known
Q8BIE6	FRMD4A	not known
Q8BJA3	HMBOX1	not known
Q8BK72	MRPS27	not known
Q8BL06	USP54	not known
Q8BL66	EEA1	not known
Q8BLN6	UNC80	not known
Q8BM13	OLFM2	not known
Q8BM92	CDH7	not known
Q8BMB3	EIF4E2	not known
Q8BML1	MICAL2	not known
Q8BNA6	FAT3	not known
Q8BNN1	SPATA2L	not known
Q8BPU7	ELMO1	not known
Q8BQM8	EML5	not known
Q8BRK8	PRKAA2	not known
Q8BSL7	ARF2	not known
Q8BTY2	SLC4A7	not known
Q8BUM6	FAM163B	not known
Q8BW41	POMGNT2	not known
Q8BX02	KANK2	not known
Q8BXL9	IFFO1	not known
Q8BZ05	ARAP2	not known
Q8BZ60	STON2	not known
Q8BZI0	AFAP1L1	not known
Q8BZZ3	WWP1	not known
Q8C008	DZANK1	not known
Q8C079	STRIP1	not known
Q8C0D9	CEP68	not known
Q8C0L0	TMX4	not known
Q8C1B1	CAMSAP2	not known
Q8C5Q4	GRSF1	not known
Q8C753	KIAA0556	not known
Q8C863	ITCH	not known
Q8C9H6	STRIP2	not known
Q8CA95	PDE10A	not known
Q8CCJ4	AMER2	not known
Q8CCN5	BCAS3	not known
Q8CDG3	VCPIP1	not known
Q8CFI7	POL2RB	not known
Q8CGM1	BAI2	not known
Q8CGU1	CALCOCO1	not known
Q8CH09	SUGP2	not known
Q8CI59	STEAP3	not known

Q8CI78	RMND1	not known
Q8CIW6	SLC26A6	not known
Q8JZR6	SLC4A8	not known
Q8JZW5	SH2D5	not known
Q8K004	SPATA2	not known
Q8K1S6	SPIRE2	not known
Q8K2J0	PLCD3	not known
Q8K2Y0	RNF219	not known
Q8K3B1	FBXO45	not known
Q8K3E5	AHI1	not known
Q8K3I4	MYRIP	not known
Q8K4P0	WDR33	not known
Q8K4P8	HECW1	not known
Q8R0G9	NUP133	not known
Q8R1G6	PDLIM2	not known
Q8R4C2	RUFY2	not known
Q8R4H2	ARHGEF12	not known
Q8R4I7	NETO1	not known
Q8R516	MIB2	not known
Q8R5C5	ACTR1B	not known
Q8VBY2	CAMKK1	not known
Q8VD04	GRIPAP1	not known
Q8VDV7	TBC1D19	not known
Q8VEH5	EPM2AIP1	not known
Q8VHI6	WASF3	not known
Q91V36	NRBP2	not known
Q91V51	TTLL1	not known
Q91V93	RHOBTB2	not known
Q91VW5	GOLGA4	not known
Q91W39	NCOA5	not known
Q91WL8	WWOX	not known
Q91XU0	WRNIP1	not known
Q91XU3	PIP4K2C	not known
Q91XX1	PCDHGC3	not known
Q91Y09	PCDHAC2	not known
Q91YD3	DCP1A	not known
Q91YK0	LRRC49	not known
Q91YM2	ARHGAP35	not known
Q91YU6	LZTS2	not known
Q91Z49	FYTTD1	not known
Q921Q7	RIN1	not known
Q922B6	TRAF7	not known
Q923G2	POLR2H	not known
Q99JN2	KLHL22	not known
Q99JX7	NXF1	not known

Q99K46	USP11	not known
Q99KI3	EMC3	not known
Q99ME2	WDR6	not known
Q99MJ9	DDX50	not known
Q99MS8	TPGS1	not known
Q99N57	RAF1	not known
Q99NH2	PARD3	not known
Q99P58	RAB27B	not known
Q99PL5	RRBP1	not known
Q9CPW4	ARPC5	not known
Q9CQ73	PKP2	not known
Q9CQF0	MRPL11	not known
Q9CRD2	EMC2	not known
Q9CYC6	DCP2	not known
Q9CZ62	CEP97	not known
Q9D067	MDM1	not known
Q9D0F3	LMAN1	not known
Q9D0L7	ARMC10	not known
Q9D1F0	RTL8B	not known
Q9D4H4	AMOTL1	not known
Q9D5T0	ATAD1	not known
Q9D832	DNAJB4	not known
Q9DB73	CYB5R1	not known
Q9DBS2	TPRG1L	not known
Q9DCP2	SLC38A3	not known
Q9EP71	RAI14	not known
Q9EP89	LACTB	not known
Q9ERE9	MAPK8IP2	not known
Q9ERR1	NDEL1	not known
Q9ERS5	PLEKHA2	not known
Q9ERU9	RANBP2	not known
Q9ERV1	MKRN2	not known
Q9ESN9	MAPK8IP3	not known
Q9JII1	INPP5E	not known
Q9JIK9	MRPS34	not known
Q9JL19	NCOA6	not known
Q9JMC3	DNAJA4	not known
Q9JMC8	EPB41L4B	not known
Q9QUR8	SEMA7A	not known
Q9QWI6	SRCIN1	not known
Q9QWV4	MLF1	not known
Q9QX11	CYTH1	not known
Q9QX47	SON	not known
Q9QXJ4	ARL10	not known
Q9QXL2	KIF21A	not known

Q9QXL8	NME7	not known
Q9QZB7	ACTR10	not known
Q9QZF2	GPC1	not known
Q9QZX7	SRR	not known
Q9R0A0	PEX14	not known
Q9R0A1	CLCN2	not known
Q9R1V4	ADAM11	not known
Q9WUN2	TBK1	not known
Q9WUQ2	PRE	not known
Q9WVI9	MAPK8IP1	not known
Q9WVK8	CYP46A1	not known
Q9WVL0	GSTZ1	not known
Q9Z0G0	GIPC1	not known
Q9Z0H3	SMARCB1	not known
Q9Z0J4	NOS	not known
Q9Z1K7	APC2	not known
Q9Z1R2	BAG6	not known
Q9Z1S3	RASGRP1	not known
Q9Z1W9	STK39	not known
Q9Z2C4	MTMR1	not known
Q9Z2D1	MTMR2	not known
Q9Z2I8	SUCLG2	not known
Q9Z2V6	HDAC5	not known
Q9Z307	KCNJ16	not known
Q9Z329	ITPR2	not known

Description

LINE-1 retrotransposable element ORF1 protein *Mus musculus*
La-related protein 1 *Mus musculus*
RNA-binding protein Raly *Mus musculus*
H15 domain-containing protein *Mus musculus*
Double-stranded RNA-binding protein Staufen homolog 2 *Mus musculus*
Nucleolar RNA helicase 2 *Mus musculus*
Zinc finger CCCH type-containing 7B *Mus musculus*
Ataxin-2 *Mus musculus*
Zinc finger RNA-binding protein *Mus musculus*
Keratin, type I cytoskeletal 18 *Mus musculus*
Vitronectin *Mus musculus*
RISC-loading complex subunit TARBP2 *Mus musculus*
Apolipoprotein A-I *Mus musculus*
Protein transport protein Sec23A *Mus musculus*
mRNA-decapping enzyme 1B *Mus musculus*
Enhancer of mRNA-decapping protein 4 *Mus musculus*
Sequestosome-1 *Mus musculus*
A-kinase anchor protein 9 *Mus musculus*
CLIP-associating protein 1 *Mus musculus*
CCR4-NOT transcription complex subunit 10 *Mus musculus*
Cleavage and polyadenylation specificity factor subunit 4 *Mus musculus*
Sorting nexin-16 *Mus musculus*
Unconventional myosin-IXa *Mus musculus*
RNA cytidine acetyltransferase *Mus musculus*
Enhancer of mRNA-decapping protein 3 *Mus musculus*
Protein Aster-A *Mus musculus*
Matrin-type domain-containing protein *Mus musculus*
ATP-dependent DNA/RNA helicase DHX36 *Mus musculus*
SPATS2-like protein *Mus musculus*
Transient receptor potential cation channel subfamily M member 7 *Mus muscu*
GRB10-interacting GYF protein 1 *Mus musculus*
Splicing factor 3B subunit 1 *Mus musculus*
ATP-dependent RNA helicase DHX30 *Mus musculus*
CCR4-NOT transcription complex subunit 11 *Mus musculus*
Nucleolar protein 56 *Mus musculus*
Interferon-inducible double-stranded RNA-dependent protein kinase activator
Fragile X mental retardation syndrome-related protein 2 *Mus musculus*
Double-stranded RNA-binding protein Staufen homolog 1 *Mus musculus*
Diacylglycerol kinase *Mus musculus*

SH3 domain-containing protein *Mus musculus*
Pleckstrin homology domain containing, family G (with RhoGef domain) memb
Cerebellar degeneration-related protein 2-like *Mus musculus*
Kelch-like protein 15 *Mus musculus*
Dedicator of cytokinesis protein 11 *Mus musculus*
Calmodulin-regulated spectrin-associated protein 1 *Mus musculus*
Taperin *Mus musculus*
Chromodomain-helicase-DNA-binding protein 7 *Mus musculus*
Tyrosine-protein phosphatase non-receptor type 3 *Mus musculus*
Rap1 GTPase-activating protein 1 *Mus musculus*
Sodium channel protein type 1 subunit alpha *Mus musculus*
Sickle tail protein *Mus musculus*
Calcium-binding mitochondrial carrier protein SCaMC-2 *Mus musculus*
Fibrinogen C domain-containing protein 1 *Mus musculus*
AT-rich interactive domain-containing protein 1A *Mus musculus*
Phosphodiesterase *Mus musculus*
3'-5' RNA helicase YTHDC2 *Mus musculus*
Janus kinase and microtubule-interacting protein 2 *Mus musculus*
Probable bifunctional methylenetetrahydrofolate dehydrogenase/cyclohydrolas
Diacylglycerol kinase *Mus musculus*
HECT and RLD domain-containing E3 ubiquitin protein ligase family member 1
Tensin 1 *Mus musculus*
PDZ domain-containing 2 *Mus musculus*
ATP-binding cassette, sub-family C (CFTR/MRP), member 4 *Mus musculus*
Ryanodine receptor 2 *Mus musculus*
AT-rich interactive domain-containing protein 1B *Mus musculus*
Tetratricopeptide repeat protein 7B *Mus musculus*
Utrophin *Mus musculus*
Protein furry homolog *Mus musculus*
SPRY domain-containing 3 *Mus musculus*
ANK_REP_REGION domain-containing protein *Mus musculus*
A kinase (PRKA) anchor protein 6 *Mus musculus*
Disks large homolog 5 *Mus musculus*
Ras association (RalGDS/AF-6) and pleckstrin homology domains 1 *Mus musc*
Transmembrane protein 132B *Mus musculus*
Amylo-1,6-glucosidase, 4-alpha-glucanotransferase *Mus musculus*
Unconventional myosin-X *Mus musculus*
Guanylate cyclase domain-containing protein *Mus musculus*
Zinc finger CCCH-type-containing 12B *Mus musculus*
Transient receptor potential cation channel, subfamily M, member 3 *Mus musc*
RUN domain-containing protein 3A *Mus musculus*
Kinesin-like protein KIFC2 *Mus musculus*
Calsequestrin-2 *Mus musculus*
Kinesin-like protein KIF3C *Mus musculus*
Mitogen-activated protein kinase kinase kinase 5 *Mus musculus*

Kinesin-like protein KIFC3 _Mus musculus_
AP-1 complex subunit beta-1 _Mus musculus_
Neutral amino acid transporter A _Mus musculus_
SRSF protein kinase 2 _Mus musculus_
Regulator of G-protein signaling 9 _Mus musculus_
Guanylate cyclase soluble subunit beta-1 _Mus musculus_
Phosphatidylinositol 4-phosphate 5-kinase type-1 gamma _Mus musculus_
ER membrane protein complex subunit 8 _Mus musculus_
Serine/threonine-protein kinase ULK1 _Mus musculus_
E3 ubiquitin-protein ligase TTC3 _Mus musculus_
Vesicle transport through interaction with t-SNAREs homolog 1B _Mus musculu_
Metaxin-2 _Mus musculus_
Protein lin-7 homolog B _Mus musculus_
cAMP-specific 3',5'-cyclic phosphodiesterase 4A _Mus musculus_
DNA-directed RNA polymerase II subunit RPB1 _Mus musculus_
Immunoglobulin superfamily member 11 _Mus musculus_
Dystrophin _Mus musculus_
Keratin, type II cytoskeletal 8 _Mus musculus_
Anion exchange protein 3 _Mus musculus_
Serine/threonine-protein kinase B-raf _Mus musculus_
Biglycan _Mus musculus_
Decorin _Mus musculus_
Kinesin-like protein KIF3A _Mus musculus_
AP-1 complex subunit mu-1 _Mus musculus_
NADPH--cytochrome P450 reductase _Mus musculus_
Glutamate receptor ionotropic, kainate 2 _Mus musculus_
Cytochrome c oxidase subunit 6A1, mitochondrial _Mus musculus_
Histone lysine acetyltransferase CREBBP _Mus musculus_
Pericentrin _Mus musculus_
Tyrosine-protein kinase JAK1 _Mus musculus_
Galactocerebrosidase _Mus musculus_
Cadherin-5 _Mus musculus_
Small conductance calcium-activated potassium channel protein 2 _Mus muscu_
Small conductance calcium-activated potassium channel protein 3 _Mus muscu_
Myocardin-related transcription factor B _Mus musculus_
Regulating synaptic membrane exocytosis protein 4 _Mus musculus_
Leucine-rich repeat and calponin homology domain-containing protein 1 _Mus_
Guanine nucleotide-binding protein G(I)/G(S)/G(T) subunit beta-2 _Mus muscu_
Kinesin-associated protein 3 _Mus musculus_
Unconventional myosin-If _Mus musculus_
Nuclear factor 1 X-type _Mus musculus_
PDZ and LIM domain protein 4 _Mus musculus_
Signal transducing adapter molecule 1 _Mus musculus_
Receptor-type tyrosine-protein phosphatase N2 _Mus musculus_
Cadherin-8 _Mus musculus_

Lysosomal-trafficking regulator *Mus musculus*
Peptidyl-glycine alpha-amidating monooxygenase *Mus musculus*
DNA-directed RNA polymerase II subunit RPB3 *Mus musculus*
Amyloid-beta A4 precursor protein-binding family A member 2 *Mus musculus*
Ephrin type-A receptor 4 *Mus musculus*
Protein enabled homolog *Mus musculus*
Potassium voltage-gated channel subfamily B member 1 *Mus musculus*
Cyclin-dependent kinase 16 *Mus musculus*
Serine/threonine-protein kinase MAK *Mus musculus*
Formin-1 *Mus musculus*
Exophilin-5 *Mus musculus*
MCG18019 *Mus musculus*
Protein TANC1 *Mus musculus*
Metabotropic glutamate receptor 2 *Mus musculus*
Cytospin-A *Mus musculus*
Protocadherin Fat 4 *Mus musculus*
Dolichyl-diphosphooligosaccharide--protein glycosyltransferase subunit STT3B
RIKEN cDNA 2410002F23 gene *Mus musculus*
NLR family member X1 *Mus musculus*
Trafficking protein particle complex subunit 10 *Mus musculus*
Neuropathy target esterase *Mus musculus*
Microtubule-associated protein 9 *Mus musculus*
Pre-B-cell leukemia transcription factor-interacting protein 1 *Mus musculus*
Apoptosis-inducing factor 3 *Mus musculus*
Trafficking protein particle complex subunit 9 *Mus musculus*
Prickle-like protein 1 *Mus musculus*
Uncharacterized protein KIAA0930 homolog *Mus musculus*
Disabled homolog 2-interacting protein *Mus musculus*
Adhesion G protein-coupled receptor B1 *Mus musculus*
Transcription factor HIVEP2 *Mus musculus*
Zinc finger SWIM domain-containing protein 8 *Mus musculus*
Serine-rich coiled-coil domain-containing protein 2 *Mus musculus*
Microtubule cross-linking factor 1 *Mus musculus*
Serine/threonine-protein kinase PAK 6 *Mus musculus*
Coiled-coil domain-containing protein 33 *Mus musculus*
Protein phosphatase 1 regulatory subunit 12C *Mus musculus*
G protein-regulated inducer of neurite outgrowth 1 *Mus musculus*
Sarcolemmal membrane-associated protein *Mus musculus*
Inhibitory synaptic factor 2A *Mus musculus*
Cyclin-dependent kinase-like 5 *Mus musculus*
Uncharacterized protein C7orf43 homolog *Mus musculus*
Ankyrin repeat domain-containing protein 34B *Mus musculus*
[Pyruvate dehydrogenase [acetyl-transferring]]-phosphatase 1, mitochondrial
Kinase suppressor of Ras 2 *Mus musculus*
Metabotropic glutamate receptor 5 *Mus musculus*

ELMO domain-containing protein 1 *Mus musculus*
Storkhead-box protein 2 *Mus musculus*
Mitoguardin 1 *Mus musculus*
E3 ubiquitin-protein ligase HERC2 *Mus musculus*
Pleckstrin homology domain-containing family G member 3 *Mus musculus*
Serine/threonine-protein phosphatase 6 regulatory ankyrin repeat subunit A *M*
Inactive tyrosine-protein kinase PRAG1 *Mus musculus*
BTB/POZ domain-containing protein KCTD16 *Mus musculus*
Unconventional myosin-XVI *Mus musculus*
C2 calcium-dependent domain-containing protein 4C *Mus musculus*
Protein cordon-bleu *Mus musculus*
Cadherin-12 *Mus musculus*
Protein SMG7 *Mus musculus*
Unconventional myosin-Ig *Mus musculus*
Protein phosphatase Slingshot homolog 2 *Mus musculus*
Casein kinase II subunit alpha *Mus musculus*
Syntaxin-binding protein 3 *Mus musculus*
TNF receptor-associated factor 3 *Mus musculus*
Reelin *Mus musculus*
ELAV-like protein 3 *Mus musculus*
Serine/threonine-protein phosphatase 2A 56 kDa regulatory subunit gamma is
Lamina-associated polypeptide 2, isoforms alpha/zeta *Mus musculus*
Adenomatous polyposis coli protein *Mus musculus*
B-cell receptor-associated protein 29 *Mus musculus*
Zinc finger protein 638 *Mus musculus*
Kinesin-like protein KIF3B *Mus musculus*
ATP-dependent RNA helicase DDX3Y *Mus musculus*
Tyrosine-protein kinase JAK2 *Mus musculus*
Striated muscle-specific serine/threonine-protein kinase *Mus musculus*
Guanine nucleotide exchange factor DBS *Mus musculus*
PAN2-PAN3 deadenylation complex subunit Pan3 *Mus musculus*
NUAK family SNF1-like kinase 1 *Mus musculus*
Vinculin *Mus musculus*
Catenin alpha-3 *Mus musculus*
Solute carrier family 13 member 5 *Mus musculus*
Uncharacterized protein KIAA1841 *Mus musculus*
Zinc finger protein 512 *Mus musculus*
Kazrin *Mus musculus*
Probable helicase with zinc finger domain *Mus musculus*
Probable global transcription activator SNF2L2 *Mus musculus*
Ankyrin repeat and BTB/POZ domain-containing protein BTBD11 *Mus musculu*
Pannexin-2 *Mus musculus*
Regulation of nuclear pre-mRNA domain-containing protein 2 *Mus musculus*
Protein WWC2 *Mus musculus*
DNA-directed RNA polymerase II subunit GRINL1A *Mus musculus*

5'-AMP-activated protein kinase subunit beta-2 *Mus musculus*
E3 ubiquitin-protein ligase TRIM37 *Mus musculus*
Melanoma-associated antigen E1 *Mus musculus*
Serine/threonine-protein phosphatase 2A 56 kDa regulatory subunit beta isof[illegible]
SWI/SNF complex subunit SMARCC2 *Mus musculus*
CBP80/20-dependent translation initiation factor *Mus musculus*
GRAM domain-containing protein 2B *Mus musculus*
Kv channel-interacting protein 4 *Mus musculus*
Membrane-associated guanylate kinase, WW and PDZ domain-containing prot[illegible]
Constitutive coactivator of peroxisome proliferator-activated receptor gamma [illegible]
GRB10-interacting GYF protein 2 *Mus musculus*
Membrane-associated phosphatidylinositol transfer protein 2 *Mus musculus*
Protein SMG5 *Mus musculus*
Serine/threonine-protein kinase TAO2 *Mus musculus*
Synemin *Mus musculus*
Protein phosphatase Slingshot homolog 1 *Mus musculus*
Phospholipid phosphatase-related protein type 4 *Mus musculus*
Tripartite motif-containing protein 46 *Mus musculus*
Myb-binding protein 1A *Mus musculus*
Endoplasmic reticulum junction formation protein lunapark *Mus musculus*
F-box only protein 10 *Mus musculus*
Elongator complex protein 1 *Mus musculus*
Serine/threonine-protein kinase MRCK beta *Mus musculus*
Protocadherin-19 *Mus musculus*
Protein phosphatase 1E *Mus musculus*
R3H domain-containing protein 2 *Mus musculus*
Nischarin *Mus musculus*
Neuron navigator 3 *Mus musculus*
Cullin-9 *Mus musculus*
Arginine-glutamic acid dipeptide repeats protein *Mus musculus*
E3 ubiquitin-protein ligase Praja-2 *Mus musculus*
Disheveled-associated activator of morphogenesis 2 *Mus musculus*
Uncharacterized protein KIAA0232 *Mus musculus*
SEC14 domain and spectrin repeat-containing protein 1 *Mus musculus*
Probable E3 ubiquitin-protein ligase DTX3 *Mus musculus*
WD repeat domain phosphoinositide-interacting protein 2 *Mus musculus*
Volume-regulated anion channel subunit LRRC8A *Mus musculus*
BTB/POZ domain-containing protein 10 *Mus musculus*
Dixin *Mus musculus*
Phospholipase DDHD1 *Mus musculus*
Death-associated protein kinase 1 *Mus musculus*
Rho GTPase-activating protein 33 *Mus musculus*
Myomegalin *Mus musculus*
Sorting nexin-32 *Mus musculus*
Sec24-related gene family, member B (S. cerevisiae) *Mus musculus*

Protein ZNF365 *Mus musculus*
CDKN2A-interacting protein *Mus musculus*
FERM domain-containing protein 4A *Mus musculus*
Homeobox-containing protein 1 *Mus musculus*
28S ribosomal protein S27, mitochondrial *Mus musculus*
Inactive ubiquitin carboxyl-terminal hydrolase 54 *Mus musculus*
Early endosome antigen 1 *Mus musculus*
Protein unc-80 homolog *Mus musculus*
Noelin-2 *Mus musculus*
Cadherin-7 *Mus musculus*
Eukaryotic translation initiation factor 4E type 2 *Mus musculus*
[F-actin]-monooxygenase MICAL2 *Mus musculus*
Protocadherin Fat 3 *Mus musculus*
Spermatogenesis-associated protein 2-like protein *Mus musculus*
Engulfment and cell motility protein 1 *Mus musculus*
Echinoderm microtubule-associated protein-like 5 *Mus musculus*
5'-AMP-activated protein kinase catalytic subunit alpha-2 *Mus musculus*
ADP-ribosylation factor 2 *Mus musculus*
Sodium bicarbonate cotransporter 3 *Mus musculus*
Protein FAM163B *Mus musculus*
Protein O-linked-mannose beta-1,4-N-acetylglucosaminyltransferase 2 *Mus m*
KN motif and ankyrin repeat domain-containing protein 2 *Mus musculus*
Intermediate filament family orphan 1 *Mus musculus*
Arf-GAP with Rho-GAP domain, ANK repeat and PH domain-containing protein
Stonin-2 *Mus musculus*
Actin filament-associated protein 1-like 1 *Mus musculus*
NEDD4-like E3 ubiquitin-protein ligase WWP1 *Mus musculus*
Double zinc ribbon and ankyrin repeat-containing protein 1 *Mus musculus*
Striatin-interacting protein 1 *Mus musculus*
Centrosomal protein of 68 kDa *Mus musculus*
Thioredoxin-related transmembrane protein 4 *Mus musculus*
Calmodulin-regulated spectrin-associated protein 2 *Mus musculus*
G-rich sequence factor 1 *Mus musculus*
Protein KIAA0556 *Mus musculus*
E3 ubiquitin-protein ligase Itchy *Mus musculus*
Striatin-interacting proteins 2 *Mus musculus*
cAMP and cAMP-inhibited cGMP 3',5'-cyclic phosphodiesterase 10A *Mus muscu*
APC membrane recruitment protein 2 *Mus musculus*
Breast carcinoma-amplified sequence 3 homolog *Mus musculus*
Deubiquitinating protein VCIP135 *Mus musculus*
DNA-directed RNA polymerase II subunit RPB2 *Mus musculus*
Adhesion G protein-coupled receptor B2 *Mus musculus*
Calcium-binding and coiled-coil domain-containing protein 1 *Mus musculus*
SURP and G-patch domain-containing protein 2 *Mus musculus*
Metalloreductase STEAP3 *Mus musculus*

Required for meiotic nuclear division protein 1 homolog *Mus musculus*
Solute carrier family 26 member 6 *Mus musculus*
Electroneutral sodium bicarbonate exchanger 1 *Mus musculus*
SH2 domain-containing protein 5 *Mus musculus*
Spermatogenesis-associated protein 2 *Mus musculus*
Protein spire homolog 2 *Mus musculus*
1-phosphatidylinositol 4,5-bisphosphate phosphodiesterase delta-3 *Mus musc[illegible]*
ORC ubiquitin ligase 1 *Mus musculus*
F-box/SPRY domain-containing protein 1 *Mus musculus*
Jouberin *Mus musculus*
Rab effector MyRIP *Mus musculus*
pre-mRNA 3' end processing protein WDR33 *Mus musculus*
E3 ubiquitin-protein ligase HECW1 *Mus musculus*
Nuclear pore complex protein Nup133 *Mus musculus*
PDZ and LIM domain protein 2 *Mus musculus*
RUN and FYVE domain-containing protein 2 *Mus musculus*
Rho guanine nucleotide exchange factor 12 *Mus musculus*
Neuropilin and tolloid-like protein 1 *Mus musculus*
E3 ubiquitin-protein ligase MIB2 *Mus musculus*
Beta-centractin *Mus musculus*
Calcium/calmodulin-dependent protein kinase kinase 1 *Mus musculus*
GRIP1-associated protein 1 *Mus musculus*
Rab-GAP TBC domain-containing protein *Mus musculus*
EPM2A-interacting protein 1 *Mus musculus*
Wiskott-Aldrich syndrome protein family member 3 *Mus musculus*
Nuclear receptor-binding protein 2 *Mus musculus*
Probable tubulin polyglutamylase TTLL1 *Mus musculus*
Rho-related BTB domain-containing protein 2 *Mus musculus*
Golgin subfamily A member 4 *Mus musculus*
Nuclear receptor coactivator 5 *Mus musculus*
WW domain-containing oxidoreductase *Mus musculus*
ATPase WRNIP1 *Mus musculus*
Phosphatidylinositol 5-phosphate 4-kinase type-2 gamma *Mus musculus*
Protocadherin gamma C3 *Mus musculus*
Protocadherin alpha C2 *Mus musculus*
mRNA-decapping enzyme 1A *Mus musculus*
Leucine-rich repeat-containing protein 49 *Mus musculus*
Rho GTPase-activating protein 35 *Mus musculus*
Leucine zipper putative tumor suppressor 2 *Mus musculus*
UAP56-interacting factor *Mus musculus*
Ras and Rab interactor 1 *Mus musculus*
E3 ubiquitin-protein ligase TRAF7 *Mus musculus*
DNA-directed RNA polymerases I, II, and III subunit RPABC3 *Mus musculus*
Kelch-like protein 22 *Mus musculus*
Nuclear RNA export factor 1 *Mus musculus*

Ubiquitin carboxyl-terminal hydrolase 11 *Mus musculus*
ER membrane protein complex subunit 3 *Mus musculus*
WD repeat-containing protein 6 *Mus musculus*
ATP-dependent RNA helicase DDX50 *Mus musculus*
Tubulin polyglutamylase complex subunit 1 *Mus musculus*
RAF proto-oncogene serine/threonine-protein kinase *Mus musculus*
Partitioning defective 3 homolog *Mus musculus*
Ras-related protein Rab-27B *Mus musculus*
Ribosome-binding protein 1 *Mus musculus*
Actin-related protein 2/3 complex subunit 5 *Mus musculus*
Plakophilin 2 *Mus musculus*
39S ribosomal protein L11, mitochondrial *Mus musculus*
ER membrane protein complex subunit 2 *Mus musculus*
m7GpppN-mRNA hydrolase *Mus musculus*
Centrosomal protein of 97 kDa *Mus musculus*
Nuclear protein MDM1 *Mus musculus*
Protein ERGIC-53 *Mus musculus*
Armadillo repeat-containing protein 10 *Mus musculus*
DUF4939 domain-containing protein *Mus musculus*
Angiomotin-like protein 1 *Mus musculus*
ATPase family AAA domain-containing protein 1 *Mus musculus*
DnaJ homolog subfamily B member 4 *Mus musculus*
NADH-cytochrome b5 reductase 1 *Mus musculus*
Tumor protein p63-regulated gene 1-like protein *Mus musculus*
Sodium-coupled neutral amino acid transporter 3 *Mus musculus*
Ankycorbin *Mus musculus*
Serine beta-lactamase-like protein LACTB, mitochondrial *Mus musculus*
C-Jun-amino-terminal kinase-interacting protein 2 *Mus musculus*
Nuclear distribution protein nudE-like 1 *Mus musculus*
Pleckstrin homology domain-containing family A member 2 *Mus musculus*
E3 SUMO-protein ligase RanBP2 *Mus musculus*
Probable E3 ubiquitin-protein ligase makorin-2 *Mus musculus*
C-Jun-amino-terminal kinase-interacting protein 3 *Mus musculus*
72 kDa inositol polyphosphate 5-phosphatase *Mus musculus*
28S ribosomal protein S34, mitochondrial *Mus musculus*
Nuclear receptor coactivator 6 *Mus musculus*
DnaJ homolog subfamily A member 4 *Mus musculus*
Band 4.1-like protein 4B *Mus musculus*
Semaphorin-7A *Mus musculus*
SRC kinase signaling inhibitor 1 *Mus musculus*
Myeloid leukemia factor 1 *Mus musculus*
Cytohesin-1 *Mus musculus*
Protein SON *Mus musculus*
ADP-ribosylation factor-like protein 10 *Mus musculus*
Kinesin-like protein KIF21A *Mus musculus*

Nucleoside diphosphate kinase 7 *Mus musculus*
Actin-related protein 10 *Mus musculus*
Glypican-1 *Mus musculus*
Serine racemase *Mus musculus*
Peroxisomal membrane protein PEX14 *Mus musculus*
Chloride channel protein 2 *Mus musculus*
Disintegrin and metalloproteinase domain-containing protein 11 *Mus musculus*
Serine/threonine-protein kinase TBK1 *Mus musculus*
Prolactin regulatory element-binding protein *Mus musculus*
C-Jun-amino-terminal kinase-interacting protein 1 *Mus musculus*
Cholesterol 24-hydroxylase *Mus musculus*
Maleylacetoacetate isomerase *Mus musculus*
PDZ domain-containing protein GIPC1 *Mus musculus*
SWI/SNF-related matrix-associated actin-dependent regulator of chromatin su
Nitric oxide synthase, brain *Mus musculus*
Adenomatous polyposis coli protein 2 *Mus musculus*
Large proline-rich protein BAG6 *Mus musculus*
RAS guanyl-releasing protein 1 *Mus musculus*
STE20/SPS1-related proline-alanine-rich protein kinase *Mus musculus*
Myotubularin-related protein 1 *Mus musculus*
Myotubularin-related protein 2 *Mus musculus*
Succinate--CoA ligase [GDP-forming] subunit beta, mitochondrial *Mus muscul*
Histone deacetylase 5 *Mus musculus*
Inward rectifier potassium channel 16 *Mus musculus*
Inositol 1,4,5-trisphosphate receptor type 2 *Mus musculus*

	Goodier et al. 2013 HEK293T	Taylor et al. 2013 and 2018 HEK293T	Moldovan and Moran 2015 Hela	Vuong et al. 2019 hESCs
	X	X	X	X
	X		X	X
	X			
	X			X
	X			X
	X		X	
				X
			X	
				X
				X
				X
				X
				X
				X
				X
				X
	X			
				X
				X
				X
ulus				X
				X
	X			
	X			
A *Mus musculus*				
X				

ɔer 1 *Mus musculus*

ɛe 2 *Mus musculus*

. *Mus musculus*

ulus

culus

s

lus
lus

musculus
lus

Mus musculus

Mus musculus

Mus musculus

soform *Mus musculus*

s

orm *Mus musculus*

ein 1 *Mus musculus*
Mus musculus

usculus

2 *Mus musculus*

ulus

ulus

s

ubfamily B member 1 *Mus musculus*

us

De Luca et al. 2023 spermatocytes	Ardeljan et al. 2020 Breast and ovarian human tumor
X	X
X	X
	X
X	
	X
	X
	X
X	
X	
X	
	X
X	
X	
X	
X	
